# Identification and evolution of a plant cell wall specific glycoprotein glycosyl transferase, ExAD

**DOI:** 10.1038/srep45341

**Published:** 2017-03-30

**Authors:** Svenning Rune Møller, Xueying Yi, Silvia Melina Velásquez, Sascha Gille, Pernille Louise Munke Hansen, Christian P. Poulsen, Carl Erik Olsen, Martin Rejzek, Harriet Parsons, Yang Zhang, Hans H. Wandall, Henrik Clausen, Robert A. Field, Markus Pauly, Jose M. Estevez, Jesper Harholt, Peter Ulvskov, Bent Larsen Petersen

**Affiliations:** 1 VKR Research Centre, Department of Plant and Environmental Sciences, University of Copenhagen, DK-1871, Frederiksberg C, Denmark; 2 Fundación Instituto Leloir and IIBBA-CONICET, Av. Patricia Argentinas 435, Buenos Aires, C1405BWE, Argentina; 3 Instituto de Fisiología, Biología Molecular y Neurociencias, IFIByNE-CONICET, Facultad de Ciencias Exactas y Naturales, Universidad de Buenos Aires, Intendente Güiraldes 2160, Ciudad Universitaria, Pabellón II, Buenos Aires, C1428EGA, Argentina; 4 Institute for Plant Cell Biology and Biotechnology, Heinrich-Heine University, Duesseldorf, Germany; 5 Carlsberg Research Laboratory, J. C. Jacobsens Gade 4, 1799, Copenhagen V, Denmark; 6 Department of Biological Chemistry, John Innes Centre, Norwich Research Park, Norwich, NR4 7UH, UK; 7 Copenhagen Center for Glycomics, Department of Molecular and Cellular Medicine and School of Dentistry, Faculty of Health Sciences, University of Copenhagen, DK-2200, Copenhagen N, Denmark

## Abstract

Extensins are plant cell wall glycoproteins that act as scaffolds for the deposition of the main wall carbohydrate polymers, which are interlocked into the supramolecular wall structure through intra- and inter-molecular iso-di-tyrosine crosslinks within the extensin backbone. In the conserved canonical extensin repeat, Ser-Hyp_4_, serine and the consecutive C4-hydroxyprolines (Hyps) are substituted with an α-galactose and 1–5 β- or α-linked arabinofuranoses (Ara*f*s), respectively. These modifications are required for correct extended structure and function of the extensin network. Here, we identified a single *Arabidopsis thaliana* gene, At3g57630, in clade E of the inverting Glycosyltransferase family GT47 as a candidate for the transfer of Ara*f* to Hyp-arabinofuranotriose (Hyp-β1,4Ara*f*-β1,2Ara*f*-β1,2Ara*f*) side chains in an α-linkage, to yield Hyp-Ara*f*_4_ which is exclusively found in extensins. T-DNA knock-out mutants of At3g57630 showed a truncated root hair phenotype, as seen for mutants of all hitherto characterized extensin glycosylation enzymes; both root hair and glycan phenotypes were restored upon reintroduction of At3g57630. At3g57630 was named Extensin Arabinose Deficient transferase, ExAD, accordingly. The occurrence of ExAD orthologs within the Viridiplantae along with its’ product, Hyp-Ara*f*_4_, point to ExAD being an evolutionary hallmark of terrestrial plants and charophyte green algae.

Protein *O*-linked glycans in animal and plant cells surround the cell where they function as a surface protectant^1^,^2^,^3^,^4^. One group of such *O*-glycoproteins in plants is the extensins, which define a subgroup of the hydroxyproline rich glycoprotein (HRGP) superfamily and incorporate characteristic β-L-arabinofuranoside repetitive glycosylation motifs^5^,^6^,^7^. Whereas the extensin genes in their entirety are not conserved, the extensin glycosylation motifs and a core glycosylation machinery are conserved throughout the green plant lineage. In terrestrial plant cell walls, extensins constitute a minor component of both structural and functional importance during wall deposition and remodeling^8^. In contrast, in certain algae such as the chlorophyte *Chlamydomonas reinhardtii*, the wall is primarily made up of extensin-type glycoproteins^9^,^10^. The consecutive proline units in the canonical extensin repeat (SPPPP) (reviewed in Lamport *et al*.^4^), which is conserved in both chlorophytes and streptophytes [charophyacean green algae (CGA) and terrestrial plants], are hydroxylated by the glycosylation-defining prolyl-4-hydroxylases (P4Hs) to yield 4-Hydroxyproline (Hyp), which are subsequently *O*-glycosylated by the glycan initiating and elongating enzymes^4^. Extensins of chlorophyte green algae are often more diverse, with the two innermost β-linked arabinofuranoses (Ara*f*s) shared with streptophytes^9^ implying that the extensin type *O*-glycosylation predates extensins of today. While the consecutive Hyps in *C. reinhardtii* are substituted with occasionally methylated l-Ara*f* and d-Galactofuranose (Gal*f*)^9^ (Fig. 1B), consecutive Hyps in CGAs and terrestrial plants are substituted with one to five Ara*f*s with the linkages β-1,4, β-1,2, β-1,2 and α-1,3^11^, with the linkage of the rare fifth Ara*f* being currently unknown^12^. A sidechain of four Ara*f* residues on Hyp (Hyp-Ara*f*_4_) has not been found in *C. reinhardtii* and we have analyzed *Ostreococcus tauri* (a prasinophyte) and some taxa of the *Pseudochloris wilhemii* complex (chlorophytes) without detecting Hyp-Ara*f*_4_ (unpublished observations). It thus appears that Hyp-Ara*f*_4_ is a hallmark of CGA and terrestrial plant extensins. In addition, the serine in the glycosylation motif may be substituted with an α-1,3-linked galactose unit^13^. Surprisingly, Hyp-Ara*f*_3_, but not Hyp-Ara*f*_1_, _−2_ or _−4_, is found in a number of proteins that do not feature the contiguous Hyp-glycosylation motif. These include small peptide hormones (reviewed in Matsubayashi 2010^14^), wall associated kinases (WAKs) (reviewed in Kohorn and Kohorn 2009^15^) and proline-rich, extensin-like receptor kinases (PERKs) (reviewed in Nakhamchik *et al*.^16^). Furthermore, Hyp-Ara*f*_1–3_ are detected in group 1 and 5 major pollen allergens^17^ which also do not comply with the contiguous Hyp definition of glycosylation sites. Elongation of Hyp-Ara*f*_3_ with α-linked arabinose in CGAs and terrestrial plants seems to be confined to clustered proline arabinosylation of extensin substrates, and regulated accordingly.

The extensin glycosylation machinery is rather well characterized. A single gene encoding serine α-1,3-galactosyltransferase (SGT)^18^ of family GT96 was recently identified. Of the 13 glycosylation defining P4Hs present in *A. thaliana*^19^ *At*P4H2, *At*P4H5 and *At*P4H13 have been implicated in hydroxylation of consecutive Pro residues of extensins^20^,^21^. Subsequent Hyp-linked arabinosylation is initiated by β-4-hydroxyproline arabinosyltransferases 1–3 (HPAT1-3)^22^ of family GT95. Three genes of family GT77, encoding the Reduced Residual Arabinose 1–3 (RRA1-3) that extend Hyp-Ara*f*_1_ to Hyp-Ara*f*_2_, were among the first β-arabinosyltransferases to be identified^20^,^23^. Shortly thereafter Xylo-EndoGlucanase 113 (XEG113), also of family GT77, was demonstrated to elongate Hyp-Ara*f*_2_ to Hyp-Ara*f*_3_^24^. The extensin specific α-arabinosyltransferase that extends Hyp-Ara*f*_3_ to Hyp-Ara*f*_4_, however, has not yet been identified.

Here we report the identification of an extensin specific α-arabinosyltransferase, named ExAD, that extends Hyp-Ara*f*_3_ to Hyp-Ara*f*_4_ and map the occurrence within Viridiplantae of ExAD orthologs and its product, Hyp-Ara*f*_4_, which appears to be hallmark of terrestrial plant and CGA cell wall extensins. The evolution of ExAD and its product Hyp-Ara*f*_4_ is addressed and discussed.

## Results

### Candidate selection for the extensin specific α-Ara*f* T activity

**Figure 1:**
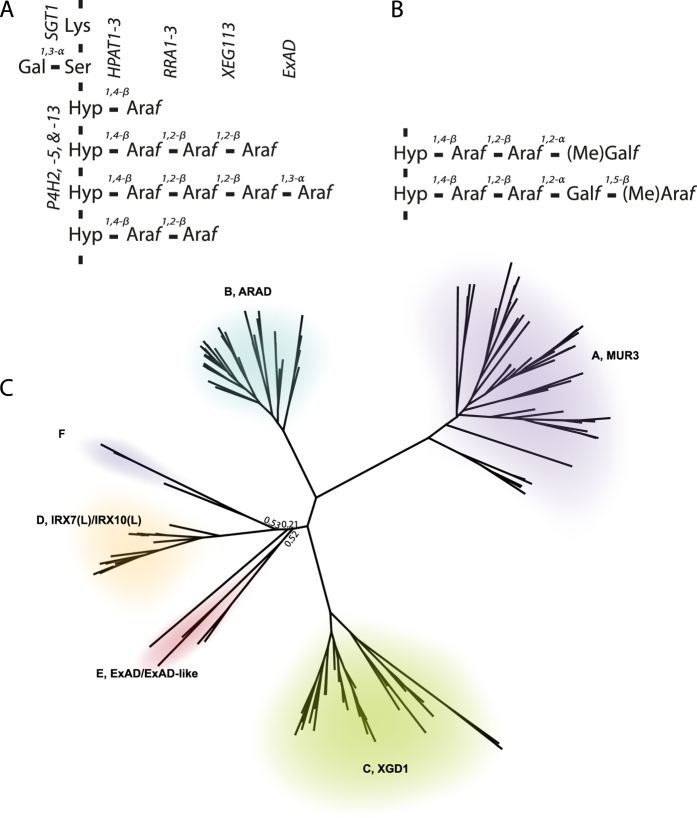
Extensin glycosylation in embryophytes and *Chlamydomonas reinhardtii* (chlorophyte). (**A**) Canonical extensin repeat structures and extensin glycosylation enzymes. In the classic extensin repeat motif, SP_3–5_^9^,^10^, serine is substituted with an α-1,3 linked galactose and the hydroxyprolines (Hyps) are substituted with β- and α-linked arabinofuranoses (Ara*f*) of lengths from 1 to 5 in terrestrial plants and CGAs. Genes encoding GTs involved in building the structures are indicated. (**B**) The innermost two Ara*f*s also exist in the chlorophyte alga *C. reinhardtii*, where they are extended by an α-1,2-galactofuranose (α-1,2Gal*f*) and a β-1,5Ara*f*, which may additionally be methylated at either the C-6 or C-3 position, respectively^9^. (**C**) Clade structure of family GT47. The tree was built from *Arabidopsis thaliana* (*At), Physcomitrella patens* (*Pp), Selaginella moellendorffii* (*Sm), Oryza sativa* (*Os)* and *Klebsormidium flaccidum*. The corresponding Newark tree file, GT47tree.txt, is provided as Supplementary Data. No known function has been demonstrated for members of the F-clade; GTs with known function, in the other clades are indicated (MUR3^61^, ARAD^62^, XGD1^63^, IRX7/IRX1^64^,^65^).

The RRA β-arabinosyltransferases in GT77 clade A were discovered by a comparative phylogenetic approach, which relied on the co-existence in this clade of terrestrial plant and *C. reinhardtii* genes, which we hypothesized to be involved in coat protein glycosylation. The co-existence of *C. reinhardtii* and plant glycosyltransferases in clade E of CAZy family GT47 led us to hypothesize that members of GT47-E were involved in extending Hyp-Ara*f*_3_ to Hyp-Ara*f*_4_ in extensins, as this particular linkage requires an inverting activity. The comparative phylogenetic approach used to identify the RRA genes relied on the fact that both *Arabidopsis thaliana* and *C. reinhardtii* feature Hyp-Ara*f*_*2*_ in their cell walls and that both species are represented in the A-clade of GT77^23^. Direct application of this approach for identifying ExAD was not feasible since *C. reinhardtii* was reported to feature neither Hyp-Ara*f*_3_ nor Hyp-Ara*f*_4_^9^ (Fig. 1).

However, our mass spectrometric analysis of *C. reinhardtii* extensin side chains additionally identified Hyp-Ara*f*_3_ together with the earlier reported structures (see Supplementary Fig. S1). This observation is corroborated by analysis of the *C. reinhardtii* genome, which implies XP_001692787.1 and XP_001690605.1 to be orthologues of XEG113, the retaining β-arabinosyltransferase of family GT77 that catalyzes the formation of Hyp-Ara*f*_3_. Nonetheless, Hyp-Ara*f*_4_ was not detected. We reasoned that ExAD might be homologous to particular Hyp-Ara*f*_2_, Hyp-Ara*f*_3_, and/or Hyp-Ara*f*_2_-Gal*f* extending GTs from *C. reinhardtii*, and focused on the E-clade of family GT47 (Fig. 1C), which comprises a single *A. thaliana* gene, At3g57630, which we selected for further analysis.

### Mutant knock out and molecular complementation links At3g57630 to arabinosylation of extensins

**Figure 2:**
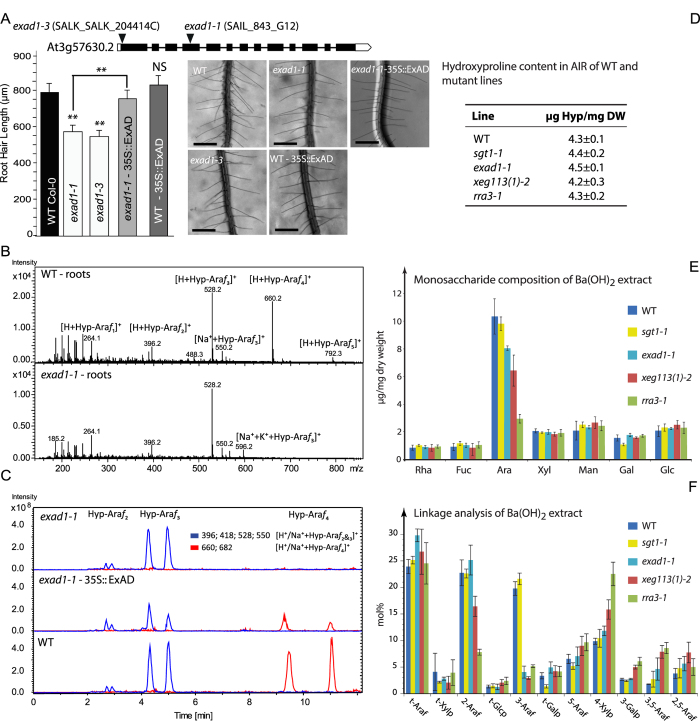
ExAD T-DNA mutant phenotypes. Root hairs of 10 days old light grown seedlings (N = 30) prepared and quantified as described in Material and Methods, NS, not significantly different, **denotes statistical significance at 1% level (student’s t-test). (**A**) Direct inlet ESI-MS of Ba(OH)_2_ hydrolysates of an extensin enriched fraction of 3 week old roots of WT and *exad1-1* (**B**). LC-ESI-MS of Ba(OH)_2_ hydrolysates of an extensin enriched fraction of 3 week old rosette leaves of *exad1-1, exad1-1* complemented with ExAD cDNA (*exad1-1*-35S::ExAD) and WT (extracted ion traces of [M + H^+^|Na^+^]^+^ 396 (Hyp-Ara*f*_2_), 528/550 (Hyp-Ara*f*_3_) and 660/682 (Hyp-Ara*f*_4_) each eluting as twin peaks due to the C-4 R/S stereo chemistry of hydroxyproline (Hyp) (**C**). For MS2 spectrum of Hyp-Ara*f*_4_ of *exad1-1***-**35S::ExAD see Supplementary Fig. S3. Hydroxyproline content (**D**), mono saccharide composition (**E**) and linkage analysis (**F**) of extensin enriched Ba(OH)_2_ extracts of 10 day old light grown seedlings of WT and the extensin glycosylation mutants (*sgt1, rra3, xeg113-2, exad1-1*).

Two T-DNA knock-out lines of At3g57630 (Supplementary Fig. S2), designated *exad1-1* and *exad1-3*, displayed a truncated root hair phenotype (Fig. 2A,B) similar to those previously reported for mutants of the extensin glycosylation-defining and elongating enzymes P4H2, P4H5, P4H13, RRA3, XEG113^20^,^21^. The root hairs were restored to WT lengths upon reintroduction of the At3g57630 cDNA under control of the CaMV 35S virus promotor in one of the mutant lines (Fig. 2A).

Direct inlet Electrospray Ionization Mass Spectrometry (ESI-MS) analysis of an extensin-enriched fraction of roots of the *exad1-1* T-DNA knock-out line subjected to Ba(OH)_2_ treatment (mediating peptide bond hydrolysis) showed the presence of Hyp-Ara*f*_1_, Hyp-Ara*f*_2_ and Hyp-Ara*f*_3_, but the complete absence of Hyp-Ara*f*_4_ (Fig. 2B,C). Again, introduction of the At3g57630 cDNA restored the Hyp-Ara*f*_4_ species (Fig. 2C). Whereas α-arabinofuranosidase treatment trimmed the Hyp-Ara*f*_4_ to Hyp-Ara*f*_3_ in the WT and *exad1-1* complemented line, Hyp-Ara*f*_1–3_ of the T-DNA mutant remained resistant, strongly suggesting that the Hyp-Ara*f*_1–3_ species of the mutant are the β-linked innermost WT Ara*f*s as anticipated (Supplementary Fig. S3).

In 10 day old seedlings the Hyp content of an extensin enriched extract of the *exad1-1, xeg113* and *rra3* mutants is unaltered compared to WT (Fig. 2D). Corresponding monosaccharide composition analysis of Ba(OH)_2_ treated extensin-enriched extracts, however, showed significant reductions in arabinose; mutants of progressively earlier-acting extensin arabinosyltransferases had an increased arabinose reduction (*xeg113, rra3*), while the *exad1-1* mutant had the smallest reduction (Fig. 2E). Galactose levels were only affected in the *sgt1* mutant, which displayed a ca 30% reduction with respect to WT levels (Fig. 2E). This substitution pattern was substantiated by linkage analysis of the extensin extracts of *xeg113, rra3* and *exad1-1*. Terminal arabinose was present at the same level in all mutants, indicating that the number of extensin arabinosyl-sidechains remained the same. The 3-linked arabinofuranosyl linkage was reduced in all three mutants to the same low level, whereas the 2-linked arabinofuranosyl linkage was not reduced in the *exad* mutant, but progressively in the *xeg113* and *rra3* mutants. These data are consistent with a role of ExAD in adding the last arabinosyl-residue at the 3 position, as shown in the model in Fig. 1A. In the *sgt1* mutant 2- and 3-linked arabinose remained at WT levels (Fig. 2F) and only terminal galactose was reduced to ca. 35% of WT level. The relative (mol %) up-regulation of 5-, 2,5- and 3,5 linked arabinose and 3-linked galactose, which are diagnostic of arabinogalactan proteins, and 4-linked xylose from xylan can be attributed to compensation of the strong decrease of 2- and 3 and linked arabinose. Hence, extensin galactosylation and arabinosylation appear to be independent. In agreement with this, the Hyp-Ara*f*_1–4_ distribution of an extensin-enriched extract of *sgt1* was found to be similar to WT, as evidenced by LC-ESI-MS analysis (data not shown).

Taken together, we conclude that At3g57630 encodes an Ara*f* T that adds the fourth α-1,3-linked Ara*f* onto Hyp-Ara*f*_3_ in classical extensin repeats. At3g57630 was named Extensin Arabinose Deficient (ExAD) after the mutant phenotype, accordingly.

### *In vitro* assays of heterologous expressed ExAD

In order to assess the substrate specificity of ExAD we produced the soluble part of the At3g57630 protein by secreted expression in *P. pastoris* and baculovirus High Five cells. Assays for arabinosyltranferase activity were conducted using chemically synthesized UDP-β-L-Ara*f* as donor substrate. Either a Hyp-Ara*f*_1–3_ mixture [obtained as a Ba(OH)_2_ hydrolysate of the mutant *exad1-1* (Fig. 2B)] or the human Mucin 1 peptide featuring Hyp-Ara*f*_3_ [expressed in Tobacco Bright Yellow 2 BY2 suspension cells^25^], were used as acceptor substrates (Supplementary Fig. S4). Neither single Hyp nor Mucin 1 peptide substituted with Ara*f*_3_ proved to be substrates. These negative results are in line with the reported extensin-type arabinosylation of endogenous and ectopically expressed substrates, where Hyp-Ara*f*_3_ appear to be necessary but not sufficient for defining the ExAD acceptor substrate.

### ExAD expression profiling

**Figure 3:**
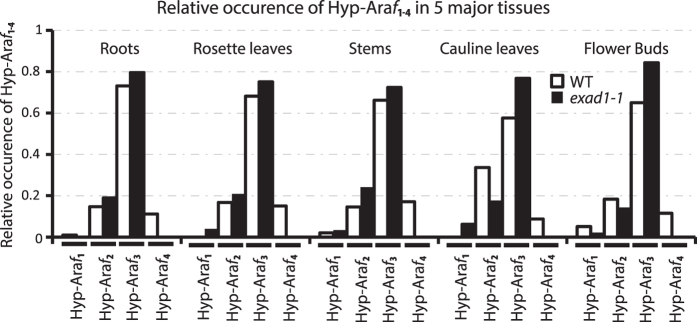
ExAD is exclusively responsible for addition of alpha linked Ara*f* on extensins. In order to access the global developmental and organotypic consequence of knocking out ExAD we prepared extensin enriched fractions from (etiolated) 7 day old seedlings (See Fig. 2) and the major tissues (roots, rosette leaves, stems, flower buds and siliques) of 6- week old bolting plants of WT and *exad1-1* and subjected these to LC-ESI-MS analysis and the extracted ion traces of [M + H^+^|Na^+^]^+^ (m/z 264 (Hyp-Ara*f*_1_), 396 (Hyp-Ara*f*_2_), 528/550 (Hyp-Ara*f*_3_) and 660/682 (Hyp-Ara*f*_4_) were semi quantified as described in the Material & Methods section and presented as the relative occurrence of each Hyp-Ara*f* specie. In all tissues investigated Hyps substituted with Ara*f*s exceeding the length of 3 were only found in WT, thus globally linking the single ExAD gene to the addition of the fourth extensin specific Ara*f* of extensins in *A. thaliana*.

Geneinvestigator Affymetrix data (https://genevestigator.com/gv/plant.jsp) suggest that At3g57630 is ubiquitously expressed at medium to high levels in all major plant tissues (Supplementary Fig. S5). We analyzed extensin-enriched fractions from 7 day old etiolated seedlings as well as the major tissues (roots, rosette leaves, stems, flowerbuds and siliques) of 6 week old bolting plants of WT and *exad1-1*; we found that Hyp-Ara*f*_4_ were present in all tissues in WT but absent in the mutant, thus unambiguously linking the single ExAD gene to the addition of the fourth extensin specific Ara*f* of extensins in *A. thaliana* (Fig. 3).

**Figure 4:**
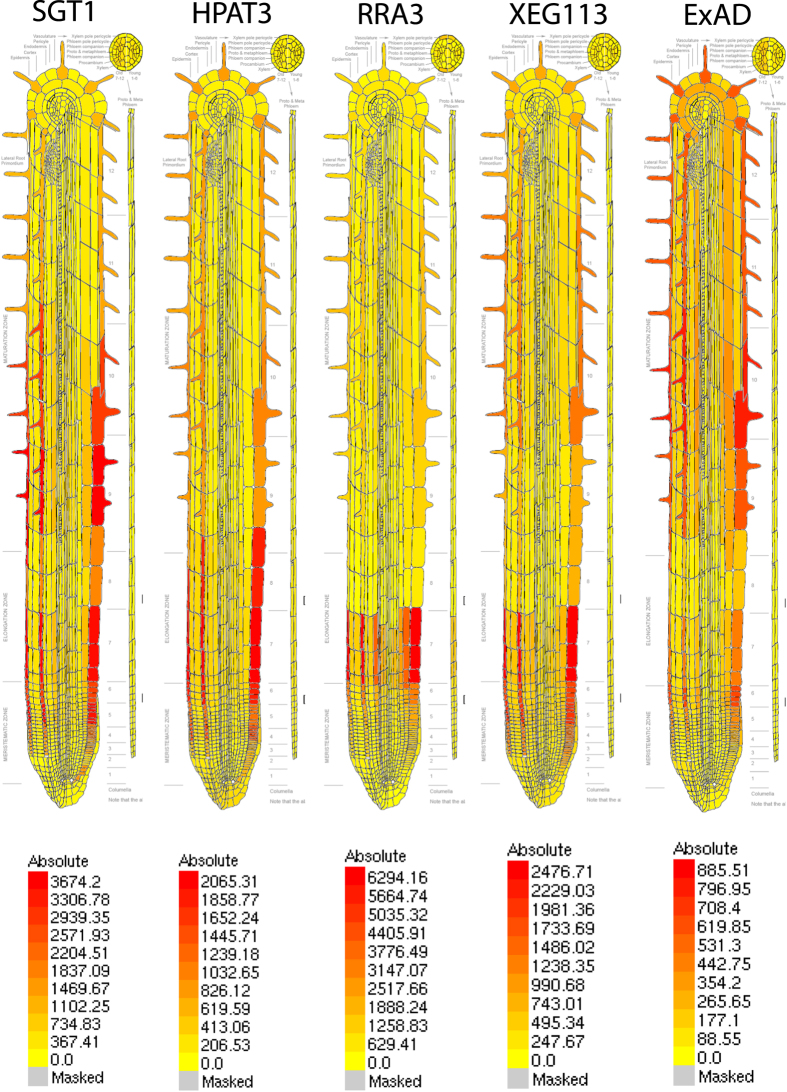
ExAD is highly expressed in root hair cells. Expression of ExAD in root epidermis and root hair cells is similar to the expression pattern of the previously characterized extensin Ara*f* Ts HPAT3^22^, RRA3^20^, XEG113^20^) and the Ser-GalT^18^ (SGT). Expression patterns were obtained using eFP-Browser^66^ (http://www.bar.utoronto.ca/) and the dataset of Brady *et al*.^51^ as described in the Material and Methods section.

This suggests that ExAD represents a ubiquitously expressed housekeeping gene. The *exad1-1* and *exad1-3* root hair mutant phenotype (Fig. 2A) is in accordance with high ExAD expression in root hair cells and with similar high expression profiles of previously characterized extensin Ara*f* Ts (HPAT3^22^, RRA3^20^, XEG113^20^) (Fig. 4) with strikingly similar expression to extensin specific Ser-GalT^18^ (SGT).

Lamport and Miller^5^ observed a higher proportion of Hyp-Ara*f*_4_ in suspension cultures of sycamore and other dicot species suggesting that Hyp-Ara*f*_3_ arabinosylation is subject to regulation. To test whether this observation is ubiquitously valid, we analyzed extensin-type glycosylation in young expanding leaves from *A. thaliana*, on an AGP fraction and on an extensin fraction from sycamore. We found Hyp-Ara*f*_4_/Hyp-Ara*f*_3_ ratios of ca 0.25 and ca. 0.7 & 1.4, respectively (Supplementary Fig. S6). This shows substantial variation in comparable tissues between species, yet the higher ratios in sycamore leaves are still substantially lower than the ratio of 4.4 observed in sycamore suspension cultures by Lamport and Miller^5^. Suspension culture cells might preferentially slough off a higher amount of soluble wall proteins to the medium and for compensation purposes up-regulate their synthesis, but the ratio was lower in this fraction when extracted from leaves. Thus, up-regulation of Hyp-Ara*f*_3_ arabinosylation in suspension cultures cannot be ruled out.

ExAD is ubiquitously expressed and there are thus limits to how strong co-expression patterns may be expected. Still, a co-expression network comprising several genes involved in extensin biosynthesis and post translational modifications suggests a tighter co-regulation of the extensin/extensin-like glycosylation enzyme machinery core (HPAT3, XEG113, SGT1, RRA3, P4H5 & -2) and ExAD being more moderately co-expressed with the core as expected. AtEXT3 and with it several other genes encoding extensin backbones, form a separate co-expression network (Supplementary Fig. S7).

### ExAD is located in the *cis* to *medial* Golgi apparatus

**Figure 5:**
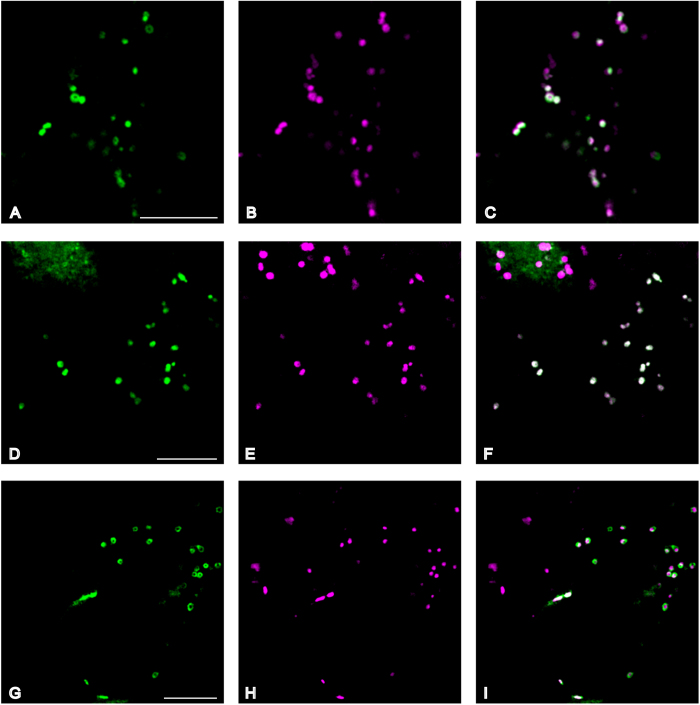
ExAD is located in the cis to medial Golgi apparatus. The full-length genomic sequence of ExAD C-terminally fused to mTurquoise3 under control of the CaMV 35S virus promoter was expressed transiently in leaves of *N. benthamiana* using agrobacterium mediated infiltration. Fluorescence of EXAD-mTurquoise3 (green) partly co-localized (white) with co-expressed known cis Golgi localized GlcNAc transferase I (GnTI) (**A**–**C**), fully with co-expressed medial Golgi localized mannosidase II (Man II) (**D**–**F**), and not with trans Golgi localized co-expressed Sialyl-Transferase (ST) (**G**–**I**) all C-terminally fused to either mRFP or YFP (Magenta) and under control of the CaMV 35S promoter. Co-localization is evident (white) in section F, which, due to the nature of the experiment (transient expression), also contains cells that solely express the marker GMII-mRFP (upper left in the section). Scale bar = 10 μm.

The full-length genomic sequence of ExAD (At3g57630) C-terminally fused to mTurquoise3 was expressed under control of the CaMV 35S promoter in leaves of *Nicotiana benthamiana* to elucidate the subcellular localization of the At3g57630 protein (Fig. 5). N-Glycan N-acetylglucosaminyltransferase I fused to monomeric red fluorescent protein (GnTI-mRFP)^26^, N-glycan α-mannosidase II fused to monomeric RFP (GMII-mRFP)^27^ and sialyltransferase fused to yellow fluorescent protein (ST-YFP)^28^ were used as markers for cis-, medial- and trans-Golgi cisternae, respectively. Fluorescence of ExAD-mTurquoise3 co-localized with medial-Golgi-targeted mannosidase II (GMII) and partly with cis-Golgi localized GlcNAc transferase I (GnTI), both under control of the CaMV 35S promoter (Supplementary Material and Methods Fig. S11). This localization in the medial part of the Golgi membrane system is in accordance with the reported location of other extensin glycosylation-defining and elongating enzymes^20^,^21^.

### ExAD protein structure

**Figure 6:**
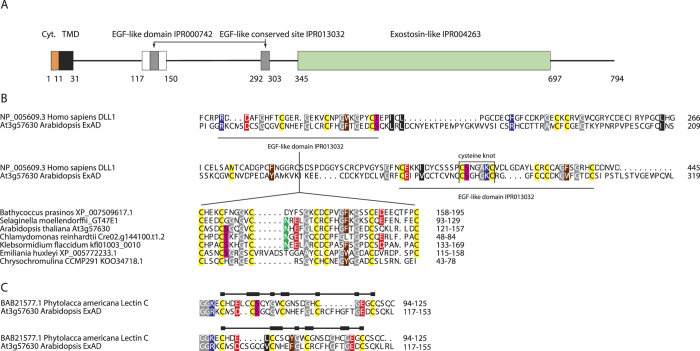
ExAD protein structure as predicted by Interproscan. (**A**) ExAD (At3g57630) is a type-II membrane anchored GT with a short (11 aa) cytoplasmic tail preceding the transmembrane domain (TMD). The catalytic domain adopts a GT-B fold and is related to the mammalian type exostosin domain found in heparan sulfate synthases^67^. The N-terminal sequence between the TMD and the catalytic domain features two putative epidermal growth factor (EGF) like domains followed by what appears to be a flexible linker. (**B**) The domains that display sequence similarity with EGF motifs align with similar domains in human delta-like 1. Alignment of the first domain in ExAD and ExAD-like sequences is shown for selected Viridiplantae and haptophyte sequences. A cysteine knot, potential substrate for fucosylation, is indicated in the second domain. (**C**) Two curated alignments of part of the ExAD N-terminal with a partial sequence of pokeweed lectin-C. This lectin binds chitin and the region critical for binding is marked by a horizontal bar and with thicker boxes indicating fully conserved residues^30^.

ExAD (At3g57630) encodes a typical Type-II membrane anchored protein of 793 amino acids, making it the largest of the 39 *A. thaliana* genes in family GT47. Our observation that a human MUC1 epitope expressed in *A. thaliana* was consistently arabinosylated to Hyp-Ara*f*_3_ and never to Hyp-Ara*f*_4_^25^ just like the peptide hormones and that two Hyp-Ara*f*_3_-containing acceptor substrates failed to work in *in vitro* ExAD assays lead us to hypothesize that the presence of Hyp-Ara*f*_3_ does not fully define ExAD’s acceptor specificity. The additional structural requirements may be probed by ExAD through protein-protein or protein-carbohydrate interactions. Examination of the longer N-terminal domain reveals a highly structured polypeptide featuring two epidermal growth factor (EGF) like domains. These are characterized by cysteine knots, which are substrates for *O*-fucosylation in mammalian Notch signaling^29^. Figure 6A shows a partial alignment of ExAD with human Delta1, a Notch signaling component with the cysteine knot of the second EGF-like domain indicated. The first EGF-like domain also resembles a highly conserved domain in Lectin C (GlcNAc binding lectin^30^) albeit with an invariant Gly residue missing.

We have also scanned full length ExAD homologous sequences, used in Fig. 1C, for domains in an unsupervised manner using SALAD^31^ (Supplementary Fig. S8) which is a comparative plant genomics motif-based database of protein annotations where similarity clustering is based on distribution patterns of such motifs in the query sequences. Interestingly both clades of sequences, including the two available full-length prasinophyte ExADs, contain EGF-like domains (domain 4 and 9) along with C-terminal exostosin-specific domains. The haptophyte sequences included in the tree feature one EGF-like domain; only in the rhodophyte *Chondrus crispus* sequence (which is a outgroup and not *bona fide* member of clade E) are the EGF-like domains completely missing. Beside the EGF-like domains in the N-terminal part, exostosin-specific domains are conserved. Obviously, the long N-terminal of the ExAD sequence is not merely an extended, flexible linker inserted between membrane anchor and catalytic domain. Rather it features highly structured domains that could recognize carbohydrate epitopes on the extensin polypeptide or serve the purpose of interacting with other protein(s) essential for its function.

### Evolution of ExAD and ExAD-like genes

The substantial number of *C. reinhardtii* genes in clade E of GT47, combined with the absence of Hyp-Ara*f*_4_ in *C. reinhardtii*, raises the question of when in evolution a GT47 clade E protein was first recruited to transfer an Ara*f* to Hyp-Ara*f*_3_ to form Hyp-Ara*f*_4_. Likewise, what sets these GTs apart from the other clade E GTs with different catalytic activities. We propose to name the Hyp-Ara*f*_4_ forming GTs ExAD and the other clade E members ExAD-like.

**Figure 7:**
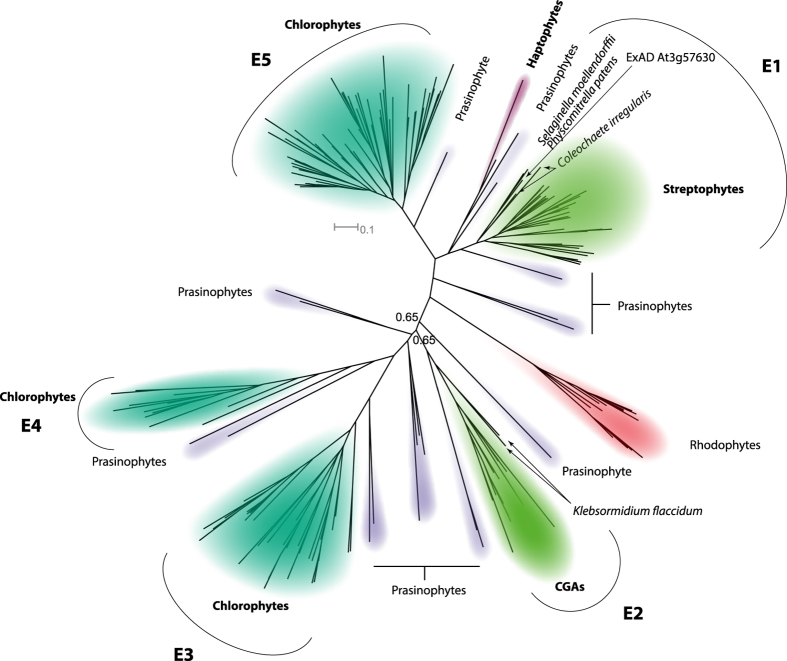
Expansion of the E-clade with prasinophyte, chlorophyte, charophyte and haptophyte sequences. Rhodophyte homologs are used as outgroup. Three chlorophyte, one pure CGA and one streptophyte sub-clade, are proposed. Subtending prasinophyte sequences are not considered included in the sub-clades. All CGAs except *Klebsormidium flaccidum* are transcripts, *i.e*. partial. *Chlamydomonas reinhardtii* has 19 clade-E sequences. Only one is indicated, the one with the highest similarity to AtExAD. The complete tree with all taxa is provided as Supplementary Fig. S9.

Figure 7 shows an expansion of the E-clade with prasinophyte, chlorophyte and charophyte sequences. Red algae were selected as an outgroup for rooting the tree, which comprises one charophyte *sensu stricto* (CGA), one streptophyte *senso lato* and three chlorophyte sub-clades for which naming are proposed. All clades feature ancestral prasinophyte sequences. Close examination (see Supplementary Fig. S9) reveals that while some charophytes are present in both the streptophyte and charophyte clades, others are present in only one of the two. Embryophytes were only found in sub-clade E1. The simplest assumption that accounts for the structure of the whole E-clade is that the ancestral organism had two ExAD-like genes, each giving rise to the clades on either side of the red algal clade. Embryophytes appear to have lost one of them and *Klebsormidium flaccidum*, the only CGA that has had its genome sequenced^32^, is only present in the opposite CGA-clade, subclade E2. It is noteworthy that none of the chlorophyte sequences were predicted to be membrane-anchored, while those of the terrestrial plants and *K. flaccidum* all appear to be type-II membrane-anchored proteins. All other CGA-sequences are transcripts from the 1 Kp-project (www.onekp.com) and none of these have complete N-terminals.

To ascertain the presence of Hyp-Ara*f*_4_ we subjected a Ba(OH)_2_ hydrolysate of *K. flaccidum* Alcohol Insoluble Residue (AIR) to LC-ESI-MS analysis.

**Figure 8:**
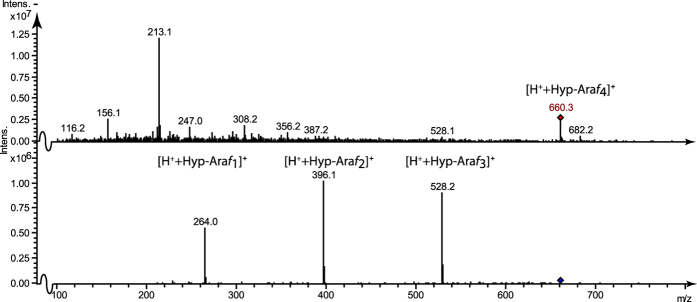
Klebsormidium flaccidum contains Hyp-Araf_4_. The MS and MS/MS spectrum of a Ba(OH)_2_ hydrolysate of *K. flaccidum* Alcohol Insoluble Residue (AIR)/cell wall fraction demonstrates the presence of Hyp-Ara*f*_4_ [H^+^ + Hyp-Ara*f*_4_]^+^.

The MS/MS spectrum in Fig. 8 demonstrates the presence of Hyp-Ara*f*_4_. Taken together with the presence of Hyp-Ara*f*_2_ and Hyp-Ara*f*_3_ as evidenced by MS/MS analysis (Supplementary Fig. S10), it appears that *K. flaccidum* has an embryophyte-type extensin arabinosylation, which is thus not confined to species with an ExAD sequence belonging in sub-clade E1. *K. flaccidum* is an early diverging CGA amongst those with a polysaccharide-based cell wall, suggesting that Hyp-Ara*f*_4_ is a general characteristic of streptophytes and thus that both the charophyte and embryophytes harbor true ExAD sequences.

## Discussion

The study of Lamport and Miller^5^ documented the occurrence of Embryophyte-type Hyp-arabinosylation as far back as mosses and liverworts, albeit with overall shorter side-chains than in higher plants. A recent bioinformatics study^33^ pointed out that only genes of vascular plants encode extensins in the strict sense. The genome of the moss *Physcomitrella patens* does not encode repeated SP_3–4_ (SP_3–4_SP_3–4_) motifs, as do the genomes of *A. thaliana*, the lycopod *Selaginella moellendorfii*, the charophycean green alga *K. flaccidum*, the chlorophyte *C. reinhardtii*, the prasinophytes *Micromonas pusilla* and *O. tauri. P. patens* and *K. flaccidum* lack the cross-linking motif SP_3–5_X_2–3_YXY that is present in the proteomes of the other species, yet wall integrity in the moss was found to rely on Hyp-arabinosylation in a recent study of knock-outs of HPAT^34^. These observations lead us to conclude that the *O*-glycosylation machinery and the glycosylation sites preceded the evolution of extensins *sensu strictu*, which should be regarded a vascular plant invention, in accordance with the study by Liu *et al*.^33^. While this would make attempts at tracking the phylogeny of extensin genes to the origin of Viridiplantae uncertain at best, this does not hold true for the arabinosylating GTs, as evidenced by the discovery of the RRAs of family GT77 and ExAD of family GT47 (present study). The *C. reinhardtii* genome comprises putative orthologs of RRAs and XEG113^35^ and it encodes several GTs belonging to family GT47, all belonging to the E-clade. It seemed reasonable that these might be involved in synthesis of the elaborate extensin-like side-chains found in its coat proteins. The extensive gene duplication would suggest functional diversification in *C. reinhardtii* so that the absence of Hyp-Ara*f*_4_ would not preclude that embryophyte orthologs could encode the Hyp-Ara*f*_3_ elongating α-arabinosyltransferase. Hence, At3g57630 was selected for characterization.

ExAD is a single copy gene in *A. thaliana* making mutant analysis promising; indeed we observed both the expected root hair phenotype, and the biochemical phenotype in terms of presence of Hyp-Ara*f*_1–3_ but not Hyp-Ara*f*_4_ in the mutant. The root hair phenotype does not imply a role of Hyp-Ara*f*_4_ confined to root hairs. Hyp-Ara*f*_4_ is present and ExAD is expressed throughout the plant. Root hairs are exposed cells in which loss of cell wall integrity readily leads to an observable phenotype.

Extensin from the Arabidopsis ExAD mutant was insensitive to α-arabinofuranosidase treatment, suggesting that it comprised exclusively β-arabinofuranosides. Complementation did not fully restore the WT phenotype, but qualitatively the expected re-emergence of Hyp-Ara*f*_4_ was observed, which could be trimmed down to Hyp-Ara*f*_3_, diagnostic of the *exad* mutant, by α–arabinofuranosidase treatment.

Our attempts to demonstrate activity of heterologously expressed ExAD *in vitro* were not successful, even though two expression hosts combined with two *a priori* relevant acceptor substrates were evaluated and UDP-Ara*f* was used as a donor substrate, thus bypassing the complexities of GT75 involvement and membrane transport. The protein structure of ExAD points to protein-protein or protein-carbohydrate interactions and precise substrate recognition is required for ExAD to distinguish between *e.g*. peptide hormones and true extensins. We thus assume that heterologously expressed ExAD was not presented with the correct acceptor or not accompanied by a required protein partner. ExAD is Golgi localized as expected and the sub-compartmental localization that we observed corroborates the notion of an assembly line for arabinosylation of extensins in the secretory pathway.

The ExAD gene product is nearly twice in size as a typical single domain GTs of family 47. The longer N-terminal extension was examined for sites for post-translational modification *e.g*. for regulatory purposes, as well as for domains suggestive of either protein-protein or protein-carbohydrate interaction. GTs with domains for carbohydrate recognition are well-known from starch biosynthesis^36^ and at least one interesting example is known from a mycobacterial arabinosyltransferase featuring a C-terminal CBM^37^. N-terminal lectin domains are observed in several plant GTs of family GT31, including the hydroxyproline-β-O-galactosyltransferase AtGALT2^38^.

The observation discussed above that endogenous peptide hormones and human mucin sequence are glycosylated with tri-arabinofuranose glycans but are not acceptor substrates for ExAD could lead to the proposition that recognition of the single galactosyl sidechain on serine would allow ExAD to discriminate extensins from other β-arabinosylated proteins. Serine galactosylation was recently shown to require C4 hydroxylated proline (O) in the ‘KSOOOO’ peptide substrate *in vitro*^18^. This suggests that galactosylation depends on hydroxylation but not on arabinosylation of the KSPPPP motif and leaves the possibility that ExAD could probe the peptide for the galactosyl residue. Knock-out of SGT1 did not lead to an altered arabinosylation pattern, including an unaltered Hyp-Ara*f*_4_ level, speaking against this. Another possibility is recognition of β-arabinofuranosides in the vicinity of the Hyp-Ara*f*_3_ is required for elongation. The ordering of extensin side-chains with respect to each other is unknown, making this question difficult to address. A domain with similarity to a conserved region in lectin C is present in the N-terminal of ExAD. The pokeweed orthologue of lectin C binds chitin, with the ‘CCS’ sequence preceding a residue that is directly involved in carbohydrate binding; this motif is strictly conserved and also found in other α-D-GlcNAc-binding proteins, such as wheat germ agglutinin and nettle agglutinin isolectin 6^30^. The ExAD sequence does not comprise this precise motif, but is anyway not expected to bind GlcNAc. However, sequences from *C. reinhardtii*, rice and *S. moellendorffii* produce similar alignments, but ExAD-like sequences from *P. patens* and micromonas do not (data not shown). Since Hyp-Ara*f*_4_ is known from both grasses and mosses we conclude that this domain is unlikely to represent a lectin domain.

Another striking feature is the occurrence of two epidermal growth factor (EGF) like domains of which the first is overlapping with the lectin C similar sequence. EGF-domains are, for example, featured in several proteins of the NOTCH signaling pathway in non-plant eukaryotes, where it is involved in cell-cell interactions^39^. EGF-domains are *e.g*. present in the apoplastic part of Wall-Associated Kinases (WAKs) involved in cell wall sensing, which bind pectin or short pectic oligogalacturonic acid fragments^15^. However, there is no homolog of the NOTCH receptor itself in *A. thaliana* clearly indicating that plants do not have a NOTCH signaling pathway analogous to that of animals. EGF features so-called cysteine knots that are fucosylated by members of CAZy-family 65 or 68^29^. The *A. thaliana* genome comprises genes classified to GT68 but not to GT65. A glucosyltransferase from family GT90 glycosylates EGF domains in Notch signaling^40^ and *A. thaliana* also has genes classified to family GT90. Sequence similarity does not prove formation of the –S-S-bridges that make up cysteine knots. Putative cysteine knots are also found in ExAD or ExAD-like sequences from *C. reinhardtii*, rice and *S. moellendorffii*. EGF-domains usually occur in many copies in apoplastic plasma membrane bound proteins among which the WAKs are typical examples. ExAD is Golgi localized and the domain occurs only twice suggesting that these highly structured protein domains have been recruited to serve a new function in ExAD. The two EGF-like domains are present in all ExAD and ExAD-like sequences from the green plant lineage, one in haptophyte sequences and none in the red algal sequences. It is noteworthy that red algae are not known to have prolyl hydroxylases and hence cannot make HRGPs. The red algal sequences homologous to ExAD thus cannot fulfill the same biological function. The observation that the two haptophytes included in the analysis have a GT47 sequence which features an EGF-like domain and which is more similar to *A. thaliana* EXAD than to any other *A. thaliana* sequence, suggests that ExAD predates the green plant lineage. Late acquisition of a metazoan EGF by horizontal gene transfer may thus be ruled out. The *K. flaccidum* proteins are minimalistic ExADs in the sense that the SALAD analysis only detected domains that are conserved in all full length ExAD sequences analyzed. The included prasinophytes are also only sharing a limited numbers of domains with core chlorophytes and plants. The differences observed in the N-terminus of ExAD and ExAD-like are distinct, but hard to link to biochemical activity, as we have only identified the activity of *At*ExAD and do not know if the ExAD-like GTs from *C. reinhardtii* are responsible for the Hyp-Ara*f*_2_-Gal*f*-Ara*f* series of extensin-like side-chains for example.

Only *Chlorokybus atmophyticus* among extant CGA species represents an earlier evolutionary state than *K. flaccidum* yet features a polysaccharide-based cell wall^41^ (*Mesostigma viride* is also charophyacean but it’s wall is made of elaborate proteinaceous scales^42^). Our observation that extensin side-chains in *A. thaliana* and *K. flaccidum* are indistinguishable may be viewed as being at odds with the conclusion above that true extensins are a vascular plant invention. The extensin repetitive sequence structure, however, prevents a precise mapping of the origin of land plant extensins and all four extensin genes of *K. flaccidum* have been classified as ‘other chimeric EXTs’^39^. The important observation is that the Hyp-Ara*f*_4_ structure must be older than the classical extensins and it may be inferred that glycosylation machinery did not evolve in step with the extensins. Prolyl hydroxylation is a shared eukaryotic phenomenon, with Hyp-Ara*f*_3_ common to the green plant lineage. Hyp-Ara*f*_4_ is characteristic of streptophytes at least as far back as *K. flaccidum*, and classical extensins with their crosslinking motifs are an embryophyte feature. The finding that the *K. flaccidum* ExADs are type-II membrane anchored proteins like those of embryophytes, yet belong to a clade E2 that comprises no vascular plants raises the question whether membrane anchoring evolved twice in the gene family. More sequence information and extensin structures from CGA families are required before is it possible to determine whether ExAD-like sequences were recruited to become true ExADs more than once and whether this recruitment is correlated with membrane anchoring.

Identification of the acceptor substrate recognized by ExAD and determining the biochemical function of the EGF-like domains are important challenges still unresolved.

## Material and Methods

### Plant Material, growth conditions and transformation of *A. thaliana*

T-DNA insertional mutants in At3g57630 (*exad1-1* (SAIL_843_G12), *exad-2* (SALK_206288C), and *exad1-3* (*SALK_204414C*) were obtained through Syngenta (SALK-204414C) and the Salk Institute (SAIL lines), respectively.

Dark grown etiolated seedlings were grown for 7 days on Murashige-Skoog-medium 0 (MS0) plates in a controlled-environment growth chamber (Percival AR-60 I, Boone, IA, USA), 20 °C and 70% relative humidity. Bolting plants of *exad1-1* &*-3* and WT *A. thaliana* ecotype Columbia (Col-0) were grown for 4–6 weeks in soil in a controlled-environment growth chamber (Percival AR-60 I, Boone, IA, USA) at a photosynthetic flux of 100–120 mol photons m^–2^ s^–1^ at 8 h light/16 h dark cycle, 20 °C and 70% relative humidity. Growth conditions for soil grown plants were essentially as described^23^.

*Agrobacterium tumefaciens* strain pGV3850 was used for agrobacterium-mediated transfomation of *A. thaliana* (ecotype Col-0) and mutant T-DNA mutant *exad1-1* (SAIL_843_G12), which was performed essentially as described by Clough and Bent (1998). In brief, Agrobacterium was transformed by electroporation and selected with appropriate antibiotics. Agrobacterium cultures were grown overnight in Luria-Bertani (LB) medium, harvested by centrifugation, and re-suspended in a 1 l buffer containing 2.3 g MS-salt (Sigma no. M-5524), 3.2 g Gamborg-5 media (Sigma no. G-2519), 50 g sucrose and 300 μl silwet-L77, Adjusted to pH 5.7 with 1 M KOH. The aerial part of bolting *A. thaliana* plants were submerged for 5 min in the bacteria suspension and allowed to dry. Scoring of primary transformants: Seeds were sterilized with 95% ethanol and 5% hypochlorite (Clough and Bent 1998) and plated on Murashige and Skoog plates (1 l H_2_O, containing 4.3 g Murashige and Skoog-salt, 10 g sucrose and 8 g agar (Scharlau no. 07004), pH 5.8) containing appropriate antibiotics stored in the dark for 2 days at 4 °C for synchronization of germination and transformants selected after 10–14 days and transferred on to soil.

### Bioinformatics

The general clade structure of family GT47 was determined as in Harholt *et al*.^43^ augmented with sequences from *Klebsormidium flaccidum* so that the clade structure now also embraces an early diverging charophycean green alga. E-clade sequences from this analysis were included in the analysis of the expanded GT47 clade E and were supplemented with sequences from full-length sequences from *C. reinhardtii, Volvox carteri, Micromonas sp*. RCC299, *Bathycoccus prasinos, Chondrus* *crispus, Emiliania huxleyi, Chrysochromulina tobin* and a large number of charophyte, chlorophyte and rhodophyte partial sequences forthcoming from the 1 Kp project^44^,^45^,^46^,^47^. The 1 Kp sequences were retrieved by BLAST as described^43^ using a cut off of 1E-25 in E value due to the shorter reads of the 1 Kp sequences. Trees were constructed on the basis of curated Muscle alignments^48^ followed by PhyML as described^35^. Identification of EGF-like domains was carried out using Interproscan^49^ and Phobius^50^ as a component of Interproscan for prediction of signal peptides and membrane anchors.

Organotypic and developmental expression profiles were obtained by use of Geneinvestigator (https://genevestigator.com/gv/plant.jsp).

The electronic Fluorescent Pictograph (eFP) – Browser available at http://www.bar.utoronto.ca/ was used to map the expression of ExAD and related genes in the root based on data published^51^.

Co-expression data and trees were obtained from ATTED (http://atted.jp).

SALAD analyses were performed as described (http://salad.dna.affrc.go.jp/salad/en/) using standard settings.

### Root hair phenotype analysis

Seedlings were germinated on agar plates in a Percival incubator at 22 °C in a growth room for 10 days at 140 μmol m^−2^ s^−1^ light intensity. For quantitative analysis of root hair phenotypes, 200 fully elongated root hairs were measured (no roots = 30) from seedlings grown on vertical plates for 10 days. Values are reported as the mean ± SD using the ImageJ 1.50 b software. Measurements on images were captured with an Olympus SZX7 Zoom microscope equipped with a Q-Colors digital camera.

### Linkage analysis and monosaccharide composition analysis of extensin extracts

Extensin extraction from etiolated seedlings (7 day old), monosaccharide composition analysis and glycosidic linkage analysis of extensin enriched Ba(OH)_2_ extracts were performed as described^24^. In brief, alcohol- insoluble residue (AIR) of the seedlings was treated with aqueous buffer, pectinases and endoglucanases to remove the matrix wall polysaccharides. The remaining residue was treated with saturated barium hydroxide (0.22 M) to solubilize the extensins. The monosaccharide composition of the extensin fraction was determined by hydrolyzing cell wall material in trifluoroacetic acid (TFA) followed by alditol acetate derivatization and GC-MS analysis. Glycosidic linkage analysis on the Ba(OH)_2_ fraction was performed by GC-MS analysis of partially methylated alditol acetates as described^52^ with modifications^53^. The hydroxyproline (Hyp) content of was determined by the colorimetric assay as described^54^.

### Subcellular localization of ExAD transiently expressed in leaves of *N. benthamiana*

DNA constructs comprising the first 77 N-terminal amino acids (CTS region) of *N. tabacum* β1,2-N-acetylglucosaminyltransferase I fused to mRFP (GnT1-mRFP)^55^ and the first 52 N-terminal amino acids of the *A. thaliana* α-mannosidase II fused to mRFP (GMII-mRFP)^55^ were kindly provided by Dr. Richard Strasser, University of Natural Resources and Life Sciences-BOKU, Austria.

Expression constructs were transformed into the *Agrobacterium tumefaciens* strain pGV3850 and Agrobacterium-mediated transient transformation of *N. benthamiana* leaves was carried out as described^56^. The Agrobacterium strains harbouring the appropriate construct (OD_600_ = 0.2) were co-infiltrated with the strain harbouring the p19 construct (OD_600_ = 0.1). Image acquisition was performed with a Leica SP5 confocal laser scanning microscope (Leica Microsystems GmbH) equipped with a 63× objective lens using CFP, YFP and RFP filter setting provided by Leica.

### Complementation and Gfp fusion constructs

The coding region of full length ExAD cDNA Genbank accession: BT011693 was obtained from Eurofins, Europe (http://www.eurofinsdna.com/ (AT3G57630-pEX-A). The primerset: FP_EXAD: CCATGGTttctcaccagaaatggaagttc, RP_EXAD_STOP: ACTAGT*tta*atgatgatgatgatgatgcttgtagtcatcatcgtccttgtagtcggaggtcttatgcagacattcttgg where capital letters designate NcoI and SpeI restriction sites, ‘*tta*’ the stop codon, underlining His_6_ and Flag (DYKDDDDK) encoding tags, using At3G5760-pEX-A as template, were used to PCR-clone the full length coding region of EXAD incl. stopcodon into open source vector pCambia 1302D (GenBankTM accession number AF234298) under the control of the Cauliflower mosaic virus 35S (CaMV-35S) promoter and the nopaline synthase gene terminator (NOS) by use of *NcoI* and *SpeI*, yielding At3g57630-F-H-pCambia 1302D.

C-terminal flag tagged exon-intron containing genomic At3g57630 (gAt3g57630-F) (Genbank accession: BT011693) was generated by PCR amplification of *A. thaliana* gDNA using the primers 5′-GGACTCTTGACCATGTTTTCTCACCAGAAATGGAAG-3′ and 5′-CTTCTCCTTTACTAGTCATTTATCGTCATCATCTTTGTAGTCGCTGGTTTTATGGAGACATTCTTG-3′ and inserted into pCambia1302D under control of the CaMV35 promotor and NOS terminator, using *SpeI* and *NcoI* and the In-Fusion cloning kit (Clontech), yielding At3g57630-F-pCambia1302D.

gAt3g57630-RFP-pCambia1302D was generated by PCR amplification of gAt3g57630-F and RFP^55^ followed by insertion into pCambia1302D using *NcoI* and *BstEII* and the In-Fusion cloning kit (Clontech). Primers for generation of gAt3g57630-RFP: 5′-GACTCTTGACCATGTTTTCTCACCAGAAATGGAAG-3′ and 5′-GCTGGTTTTATGGAGACATTCTTG-3′, using gAt3g57630-F-pCambia1302D as template, and 5′-CTCCATAAAACCAGCCCTAGGATGGCCTCCTCCGAGGAC-3′ and 5′-ATTCGAGCTGGTCACTTATCTAGAGGCGCCGGTGGAGTG-3′ using RFP^55^ as template.

gAt3g57630-mTurquoise-pCambia1302D, gAt3g57630-YFP-pCambia1302D and gAt3g57630-CFP-pCambia1302D were made by PCR amplification of mTurquoise, CFP and YFP followed by replacement of RFP in gAt3g57630-RFP-pCambia1302D using AvrII and XbaI. Primers for generation of mTurquoise: 5′-TAAAACCAGCCCTAGGATGGTGTCGAAGGGCGAG-3′ and 5′-TGGTCACttATCTAGTTACTTGTACAGTTCGTCCATGC-3′ using mTurquoise2 (obtained from Genscript) as template. Primers for generation of YFP & CFP: 5′-TAAAACCAGCCCTAGGGTGAGCAAGGGC-3′ and 5′-TGGTCACttATCTAGTTAAGCGTAATCTGGAACATCGTATG-3′ using pEarlygate101^57^ & mCerulean3^58^ as template, respectively.

PCR was performed in 25 μl reaction volumes using the ClonAMP HiFi Master Mix system (Clontech) with the cycle parameters: 3 min at 98 °C followed by 30 cycles of 30 sec at 98 °C, 30 sec at 58 °C and 30 sec at 72 °C followed by 5 min at 72 °C.

Construct designs are visualized in Supplementary Material and Methods Fig. S11.

### Isolation of Extensin enriched cell wall fractions

Alcohol insoluble residues (AIR) were isolated as described^59^ with few modifications. Briefly, plant material was harvested and frozen in liquid nitrogen. The tissue was then homogenized using a Retschmill machine (model MM200, Retsch, Haan, Germany) at 25 Hz for 1 min. The ground tissue was then suspended in 70% ethanol, vortexed, and pelleted by centrifugation at 10,000 × g for 10 min. The ethanol was decanted, and this procedure was then repeated using chloroform: methanol (1:1, vol/vol), until all chlorophyll was removed. The pellet was then washed twice in acetone. The remaining pellet was dried under vacuum for 5 min and either processed directly or stored until further use. AIR was subjected to sequential enzymatic treatment using pectin methylesterase and endo-polygalacturonanase followed by endo-cellulase (EGII; Megazyme) as described^20^,^23^, resulting in the EXT enriched cell wall fraction.

### Barium hydroxyide mediated peptide bond hydrolysis

EXT enriched cell wall fractions or pre and post assay arabinosylated Gf(Muc1-2.2TR)p were subjected to Ba(OH)_2_ mediated hydrolysis, by dissolving it in 100 μl 0.22 M Ba(OH)_2_, incubated at 108 °C overnight, spun 10 min at 12000 × g and the supernatant transferred to a new Eppendorf tube and carefully neutralized using H_2_SO_4_. The precipitate was pelleted by centrifugation 12000 × g, 10 min. The Supernatant was used as acceptor substrate and subjected to (LC)-ESI-MS analysis.

Expanding leaves of more (*Acer pseudoplatanus*) were processed similarly albeit in larger scale and with a step introduced for the isolation of loosely bound cell wall proteins presumed to include AGPs. In brief: 10 g of AIR prepared as above was stirred for 1 h in 100 mL 0.2 M CaCl_2_. The solution was dialysed against water, freezedried and then hydrolysed in saturated Ba(OH)_2_ at 108 °C overnight. The solids were washed, resuspended in 150 mL 40 mM Na-oxalate pH = 4.8 and treated with endo-PG1 as described^60^ and destarched following the protocol of the same paper. The washed and freezedried residue was subjected to Ba(OH)_2_ hydrolysis as above.

### Direct inlet and Liquid Chromatography (LC) Electrospray ionization mass spectrometry (ESI-MS)

LC-ESI-MS (3 μl supernatant injected) was carried out using an Agilent 1100 Series LC (www.agilent.com) coupled to a Bruker HCT Ultra ion trap mass spectrometer (www.bruker.com). The column was a Phenomenex Luna C8(2) (3 microM, 100A, 150 × 2.0 mm; www.phenomenex.com) preceded by a Phenomenex Gemini C18 SecurityGuard (4 × 2 mm). The oven temperature was maintained at 35 °C. The mobile phases were A, water; B, acetonitrile, both with 0.1% (v/v) HCOOH, and the flow rate was 0.2 mL min^−1^. The gradient was: 0 to 2 min, isocratic 1% B; 2 to 8.5 min, linear gradient 1 to 3% B; 8.6 to 9.6 isocratic 99% B; 9.7 to 17 min, isocratic 1% B. The mass spectrometer was run in positive electrospray mode.

Selected ion traces were semi-quantified in accordance the instructions of and using the software Bruker HCT Compass Data analysis 4.0 software.

## Additional Information

**How to cite this article:** Moeller, S. R. *et al*. Identification and evolution of a plant cell wall specific glycoprotein glycosyl transferase, ExAD. *Sci. Rep.* **7**, 45341; doi: 10.1038/srep45341 (2017).

**Publisher's note:** Springer Nature remains neutral with regard to jurisdictional claims in published maps and institutional affiliations.

## Supplementary Material

Supplementary Information

Supplementary Dataset 1

Supplementary Dataset 2

Supplementary Dataset 3

Supplementary Dataset 4
