# Corrigendum: Identification and evolution of a plant cell wall specific glycoprotein glycosyl transferase, ExAD

**DOI:** 10.1038/srep46774

**Published:** 2017-05-23

**Authors:** Svenning Rune Møller, Xueying Yi, Silvia Melina Velásquez, Sascha Gille, Pernille Louise Munke Hansen, Christian P. Poulsen, Carl Erik Olsen, Martin Rejzek, Harriet Parsons, Zhang Yang, Hans H. Wandall, Henrik Clausen, Robert A. Field, Markus Pauly, Jose M. Estevez, Jesper Harholt, Peter Ulvskov, Bent Larsen Petersen

Scientific Reports 7: Article number: 45341; 10.1038/srep45341 published online: 03 30 2017; updated: 05 23 2017.

The original version of this Article contained an error in the spelling of the author Zhang Yang, which was incorrectly given as Yang Zhang.

The Author Contributions Statement,

S.R.M., X.Y., S.M.V., S.G., P.L.M.H., C.P.P., C.E.O., M.R., H.P., Y.Z., J.H., P.U., B.L.P. conducted the experiments. H.H.W., H.C., R.A.F. assisted with data analysis. M.P., J.M.E., J.H., P.U., B.L.P. drafted the manuscript. All authors reviewed the manuscript. Svenning Rune Møller (S.R.M.), Xueying Yi (X.Y.), Silvia Melina Velásquez (S.M.V.), Sascha Gille (S.G.), Pernille Louise Munke Hansen (P.L.M.H.), Christian P. Poulsen (C.P.P.), Carl Erik Olsen (C.E.O.), Martin Rejzek (M.R.), Harriet Parsons (H.P.), Yang Zhang (Y.Z.), Hans H. Wandall (H.H.W.), Henrik Clausen (H.C.), Robert A. Field (R.A.F.), Markus Pauly (M.P.), Jose M Estevez (J.M.E.), Jesper Harholt (J.H.), Peter Ulvskov (P.U.), Bent Larsen Petersen (B.L.P.).

now reads:

S.R.M., X.Y., S.M.V., S.G., P.L.M.H., C.P.P., C.E.O., M.R., H.P., Z.Y., J.H., P.U., B.L.P. conducted the experiments. H.H.W., H.C., R.A.F. assisted with data analysis. M.P., J.M.E., J.H., P.U., B.L.P. drafted the manuscript. All authors reviewed the manuscript. Svenning Rune Møller (S.R.M.), Xueying Yi (X.Y.), Silvia Melina Velásquez (S.M.V.), Sascha Gille (S.G.), Pernille Louise Munke Hansen (P.L.M.H.), Christian P. Poulsen (C.P.P.), Carl Erik Olsen (C.E.O.), Martin Rejzek (M.R.), Harriet Parsons (H.P.), Zhang Yang (Z.Y.), Hans H. Wandall (H.H.W.), Henrik Clausen (H.C.), Robert A. Field (R.A.F.), Markus Pauly (M.P.), Jose M Estevez (J.M.E.), Jesper Harholt (J.H.), Peter Ulvskov (P.U.), Bent Larsen Petersen (B.L.P.).

This has now been corrected in both the PDF and HTML versions of the Article.

