# Supplementary material for: Identification and evolution of a plant cell wall specific glycoprotein glycosyl transferase, ExAD
Source: Sci Rep. 2017 Mar 30;7:45341. doi: 10.1038/srep45341 (PMC5371791; doi:10.1038/srep45341)
Supplement: Supplementary Information [file srep45341-s1.pdf]

Identification and evolution of a plant cell wall specific glycoprotein glycosyl transferase, ExAD

Svenning Rune Moeller, Xueying Yi, Silvia Melina Velásquez, Sascha Gille, Pernille Louise Munke Hansen, Christian Peter Poulsen, Carl Erik Olsen, Martin Rejzek, Harriet Parsons, Yang Zhang, Hans Heugh Wandall, Henrik Clausen, Robert A. Field, Markus Pauly, Jose M Estevez, Jesper Harholt, Peter Ulvskov, Bent Larsen Petersen*

*Corresponding author,

Email: blp@plen.ku.dk, Phone: +45 35322100

Supplementary figure files

Fig. S1: Mass spectrum of a barium hydroxide hydrolysate of *Chlamydomonas reinhardtii* AIR
Fig. S2: PCR genotyping of T-DNA mutant lines
Fig. S3 LC-MS analysis of α-arabinofuranosidase treated *exad1-1* complemented with ExAD (*exad1-1* - 35S::*ExAD*)
Fig. S4: Insect & yeast cell expressed ExAD assayed with two Hyp-Araf_3 substrates
Fig. S5: ExAD is expressed evenly across most tissues and organs
Fig. S6: Hyp-Ara$f_{4/3}$ ratios in sycamore young expanding leaves
Fig. S7: Co-expression network
Fig. S8: SALAD analysis of chlorophyte and streptophyte full length EXAD and EXAD-like sequences
Fig. S9: Key to the sequences used for building the phylogenetic tree in Fig. 7
Fig. S10: MS/MS spectrum of a barium hydroxide hydrolysate of *Klebsormidium accidum* AIR
Fig. S11: Construct designs

Supplementary dataset files

Newark tree file	**GT47tree.txt**<br>Newark tree that produces Fig 1C
Newark tree file	**ExpandedCladeEnewarkTree.txt**<br>Newark tree that produces Supplementary Fig. S7
Excel file	**ExpandedCladeEsequences.xlsx**<br>Translates numbers in Supplementary Fig S7 to fasta-headers
Fasta-file	**ExpandedCladeE.fasta**<br>Provides Sequence information under the fasta headers given in ExpandedCladeEsequences.xlsx

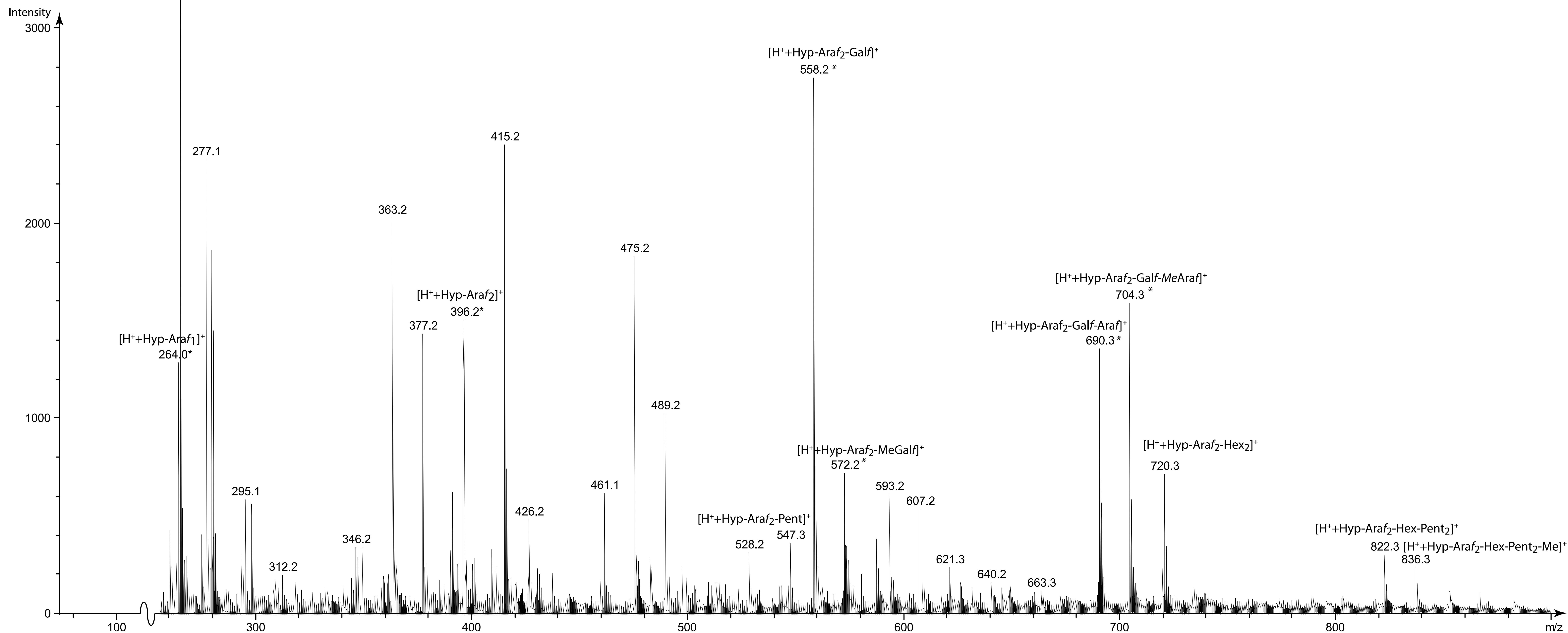

Supplementary figure S1. Mass spectrum (direct inlet) of a barium hydroxide hydrolysate of *Chlamydomonas reinhardtii* AIR. m/z values indicated with an asterix refer to published structures[12], and their annotations are given accordingly. Other m/z peaks are annotated tentatively, i.e. without providing the identity of pentosyl or hexosyl residues.

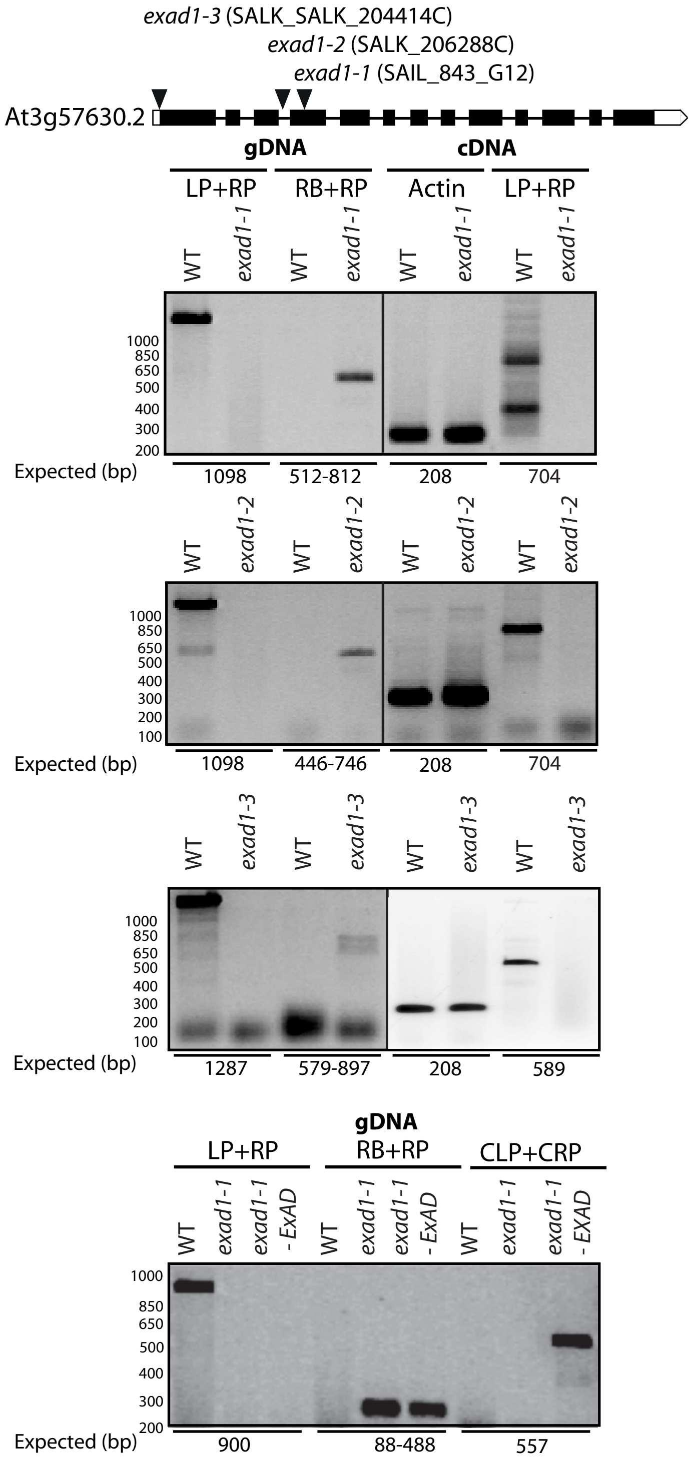

Supplementary figure S2. PCR genotyping of T-DNA mutant lines. PCR genotyping of T-DNA mutant lines *exad1-1* (SAIL_843_G12), *exad1-2* (SALK_206288C) and *exad1-3* (SALK_204414C). LP, RP and RB designate ExAD (At3g57630) gene specific Left and Right Primer and T-DNA specific Right Border primers , respectively, with LB3 and LBb1.3 used as RB for SAIL and SALK lines, respectively (http://signal.salk.edu/tdnaprimers.2.html). Below the gels, the expected PCR fragment sizes are given. LP, RP, RB, CLP (gene specific Complemented Left Primer), CRP (gene specific Complemented Right Primer) anc Actin primer sequences, gDNA isolation, cDNA synthesis and PCR procedures are provided in Supplementary Material and Methods. Construct overview is provided in Supplementary Material and Methods figure S11.

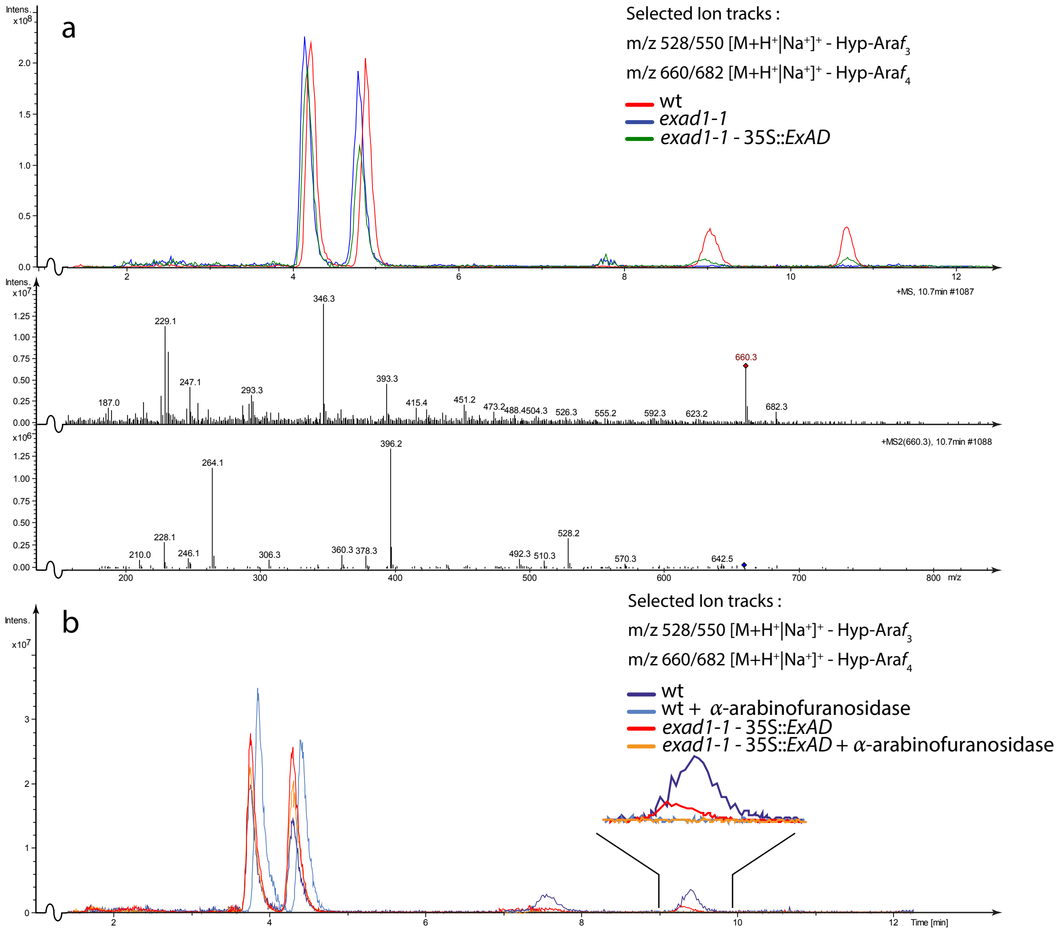

Supplementary figure S3. LC-MS analysis of α-arabinofuranosidase treated *exad1-1* complemented with ExAD (*exad1-1* - 35S::*ExAD*). *a)* selected ion-track for Hyp-Araf$_3$ ([M+H$^+$|Na$^+$]$^+$ (m/z 528/550)) and Hyp-Araf$_4$ ([M+H$^+$|Na$^+$]$^+$ (m/z 660/682)) (upper pannel) with MS2 spectrum of Hyp-Araf$_4$ of *exad1-1* complemented with *ExAD* (lower pannels). *b)* Selected ion track of Hyp-Araf$_3$ and Hyp-Araf$_4$ showing the specific removal of the Hyp-Araf$_4$ peak [H$^+$+Hyp-Araf$_4$]$^+$ (m/z 660/682) and the retainment of the Hyp-(β-Araf)$_3$ peak in the *exad1-1* complemented with ExAD + α-arabinofuranosidase combination. α-arabinofuranosidase treatment with subsequent purification is described in the Material and Methods section. Out of 8 primary complementation transformants, 3 showed biochemichal complementation with somewhat similar levels. One of the three lines is presented in panel a+b.

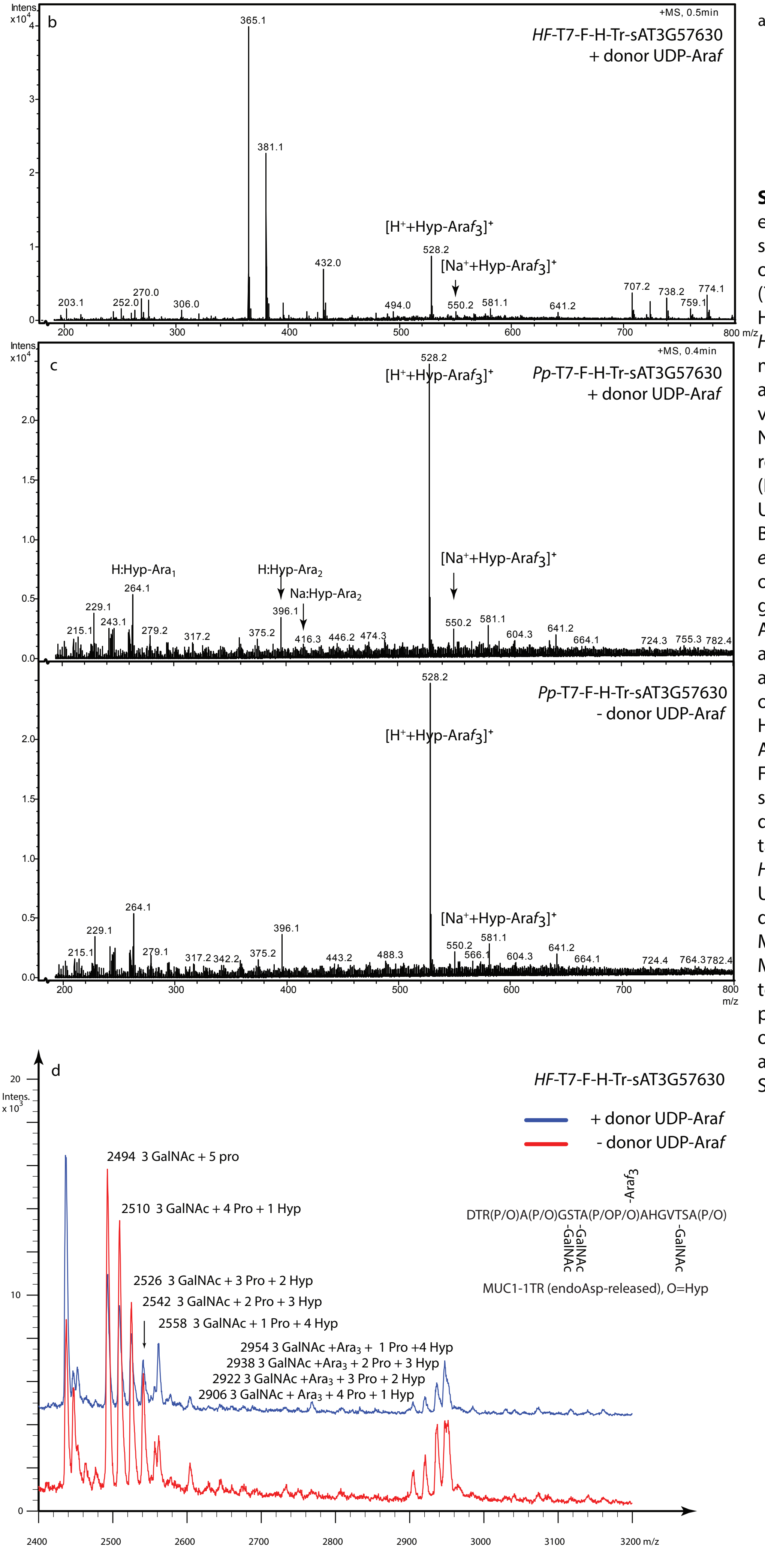

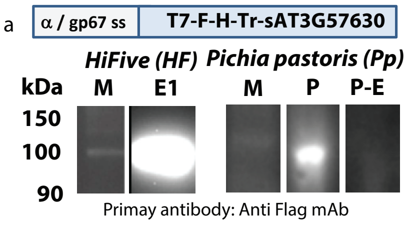

Supplementary figure S4. Insect & yeast cell expressed ExAD assayed with two Hyp-Araf_3 substrates. *a.* Western blot analysis of expression of soluble N-terminally tagged AT3G57630 (T7-F-H-Tr-sAT3G57630) in *Pichia pastoris (Pp)* and Hi Five™ (*HF*) insect cells showed that while *HF*-T7-F-H-Tr-sAT3G57630 accumulated in the media (M) fraction *Pp*-T7-F-H-Tr-sAT3G57630 accumulated inside the cells (cell pellet (P) (empty vector Ctrl pPicZαA Ctrl (P-E).
Ni-NTA purified *HF*-T7-F-H-Tr-sAT3G57630 (E1) and resuspended pellet of *Pp*-T7-F-H-Tr-sAT3G57630 (P) were assayed with or without 100 μM UDP-β-L-Araf donor substrate and with either a Ba$(OH)_2$ treated extensin enriched fraction of *exad1-1* (Hyp-Araf_{1-3}) or 2.5 Tandem Repeats (TRs) of Mucin 1, embedded in to Gfp, where each TR is glycosylated with a single arabinofuranoside of 3 Arafs in length (Gf-Muc1-2.5TR(Hyp-Araf_3)-p)[30] as acceptor substrates (d). Assay mixtures were analyzed by either ESI-MS (*b-c*) for the occurrence of Hyp-Araf_3 ($[M+H^+|Na^+]^+$ (m/z 528/550)) and Hyp-Araf_4 ($[M+H^+|Na^+]^+$ (m/z 660/682)) or Matrix Assisted Laser Desorption Ionization – Time of Flight (MALDI-TOF) mass spectroscopy of target substrate TRs, released by Endoprotease asp-N digestion, respectively, with, however, no detectable activity (MALDI-TOF data only shown for *HF*-T7-F-H-Tr-sAT3G57630). A 10 fold increase of UDP-β-L-Araf (1 mM) did likewise not yield detectable activity (data not shown). Calculated MW of Gf-Muc1-2.5TR-p is 34.360 Da[30] with the Muc1TR(Hyp-Araf_3) epitope in the assays estimated to be ca. 4 μM. O = Hyp; Araf_3 is placed at the presumed substitution site 'AOOA'. Assay conditions, Endoprotease Asp-N digestion, MALDI-TOF and ESI-MS were done as described in the Supplementary Materials and Methods.

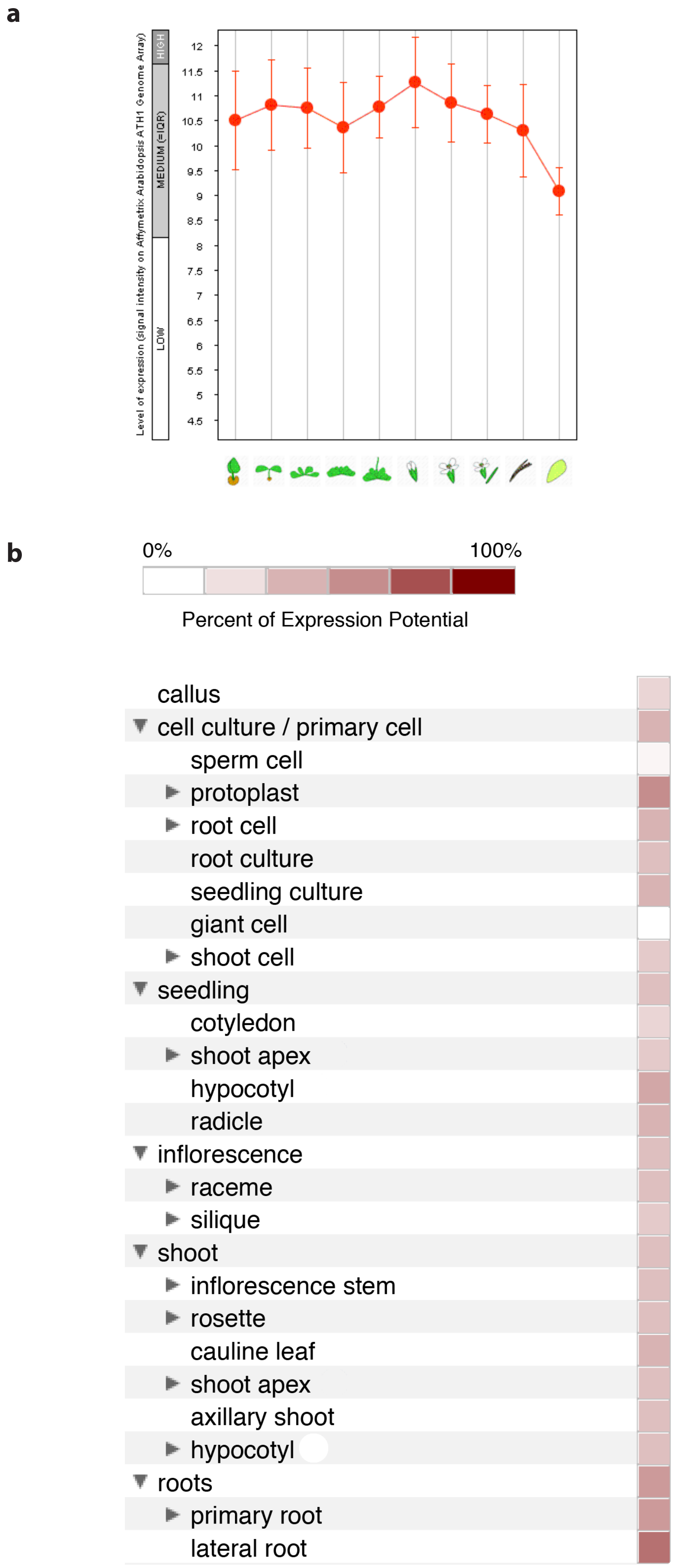

Supplementary figure S5. ExAD is expressed evenly across most tissues and organs. *a)* analysis of ten major organs and *b)* summary of measurements in 127 organs and tissues. At organo typic level high level expression is found in root tissues, e.g. roothairs (*b*, see also Figure 4), and early seed developmental tissues with medium level expression in the residual tissues (GeneInvestigator[1], https://genevestigator.com/gv/plant.jsp).

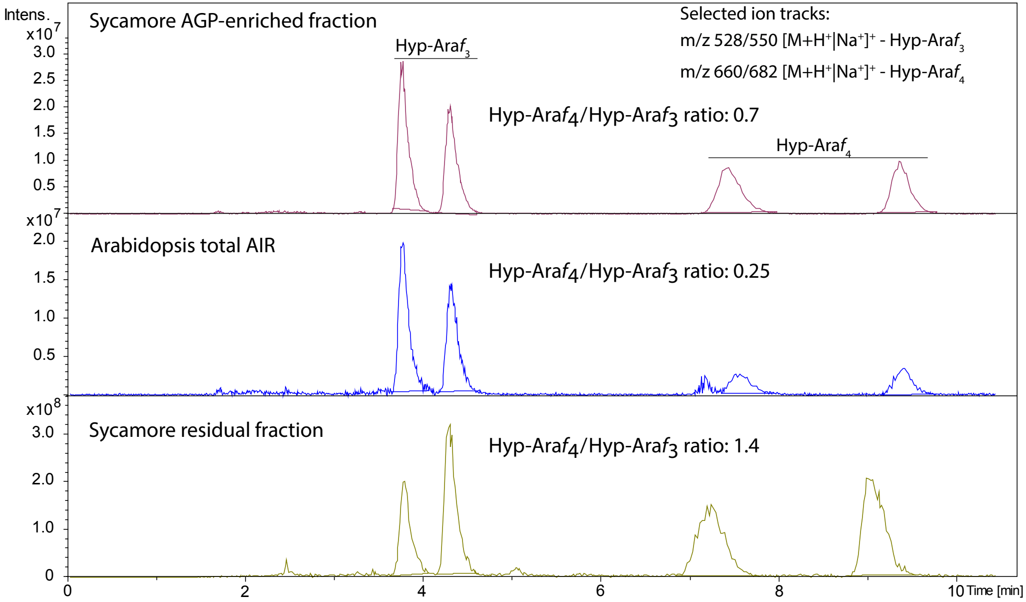

Supplementary figure S6. Hyp-Ara$f_{4/3}$ ratios in sycamore young expanding leaves. AIR of expanding leaves of sycamore, *Acer pseudoplatanus*, was extracted with 0.2 M $CaCl_2$ to obtain a readily soluble fraction of cell wall proteins assumed to comprise non-cross-linked extensins including proteins with both AGP and extensin domains. This and the residual fraction were analysed Hyp-Ara$f_{4/3}$ ratio using arabidopsis rosette AIR as a reference.

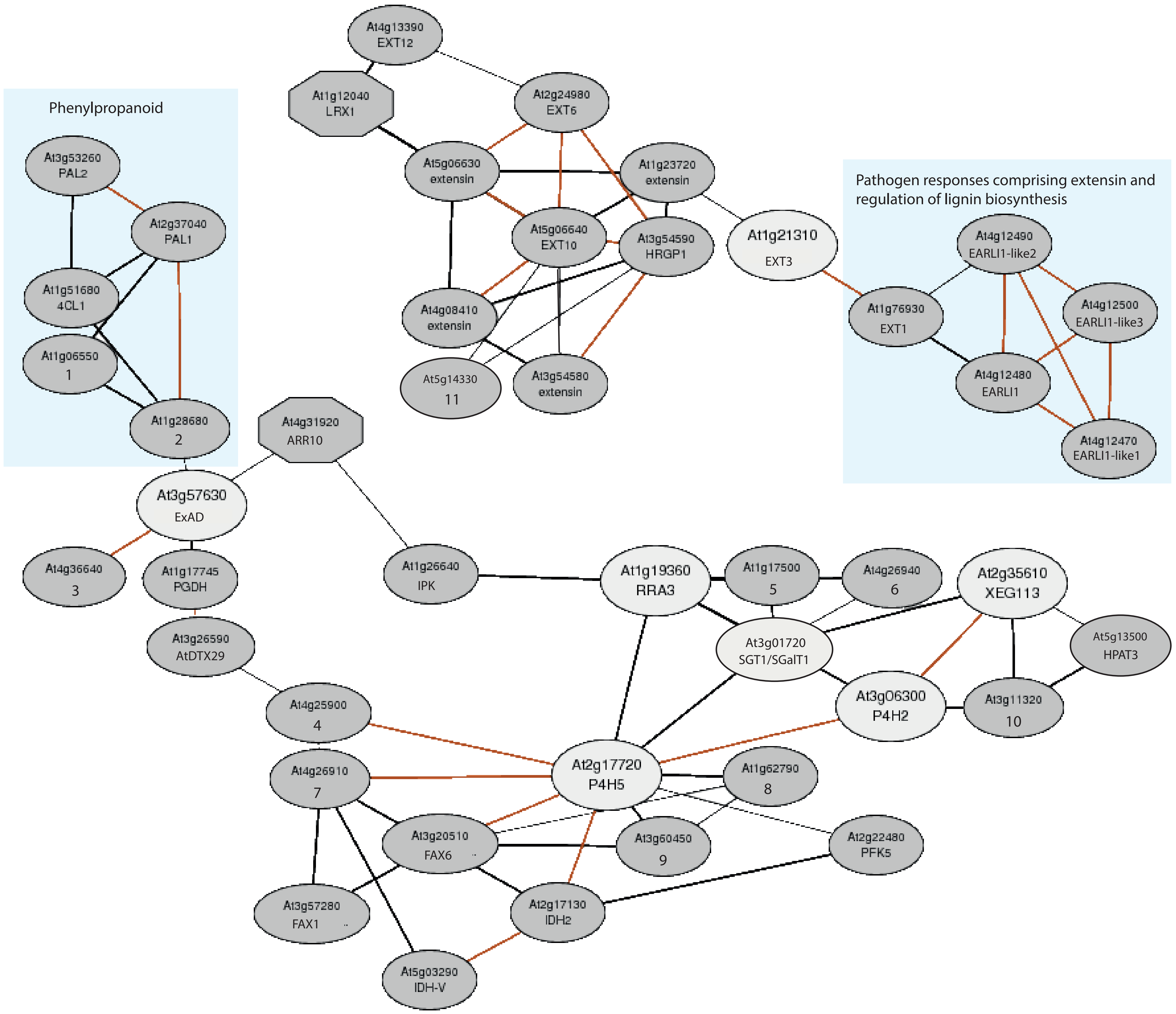

Supplementary figure S7. Co-expression network. Gene loci shown in light grey ovals were submitted to ATTED[2] to address the question whether genes encoding extensins were co-regulated with genes encoding enzymes involved in posttranslational modification of the extensins. These comprise the prolyl-4-hydroxylases represented by P4H5 and P4H2 and the GTs represented by SGT1, RRA3, XEG113 and ExAD. HPAT3, one of the arabinosyltransferases adding the innermost residue of extensin arabinosides, is a member of the network and directly connected to XEG113. ExAD is member of the same network but has no direct links to other genes encoding enzymes involved in PTM unless the acyltransferase labeled 2 and its cluster of genes shown in the box entitled "Phenylpropanoid" are to be considered involved in PTM. EXT3 was used as bait for the extensin protein backbones and indeed it has pulled in several other extensin genes but in a separate network. Part of that network comprises genes involved in reponses to injury and pathogen attack, where involvement of extensins is well documented[3]. The EARLI1 and EARLI1-like genes that cluster with EXT1 encode proline-rich proteins involved in regulation of lignin deposition during pathogen attach[4,5]. EARLI-like1 is O-glycosylated on Hyp[6] and the role of the proline rich region appears to be in subcellular localisation[7].

Unnamed genes labeled with numbers are annotated as follows:
1 At1g06550: Uncharacterized protein, best match: 3-hydroxyisobutyryl-CoA hydrolase
2 At1g28680: Uncharacterized protein, putative HXXXD-type acyl-transferase
3 At4g36640: Uncharacterized protein , Sec14p-like phosphatidylinositol transfer family protein.
4 At4g25900: Uncharacterized protein: Galactose mutarotase-like superfamily protein
5 At1g17500: Probable phospholipid-transporting ATPase 4.
6 At4g26940: Uncharacterized protein: Putative galactosyltransferase family protein from CAZy fam. 31
7 At4g26910: Putative dihydrolipoyllysine-residue succinyltransferase component of 2-oxoglutarate dehydrogenase complex 2, mitochondrial.
8 At1g62790: Uncharacterized protein: Receptor serine/threonine kinase. Bifunctional inhibitor/lipid-transfer protein/seed storage 2S albumin superfamily protein
9 At3g60450: Uncharacterized protein: Phosphoglycerate mutase family protein.
10 At3g11320: Uncharacterized protein: Nucleotide-sugar transporter family protein (UDP-carbohydrate transport)
11 At5g14330: Uncharacterized protein: Unknown protein.

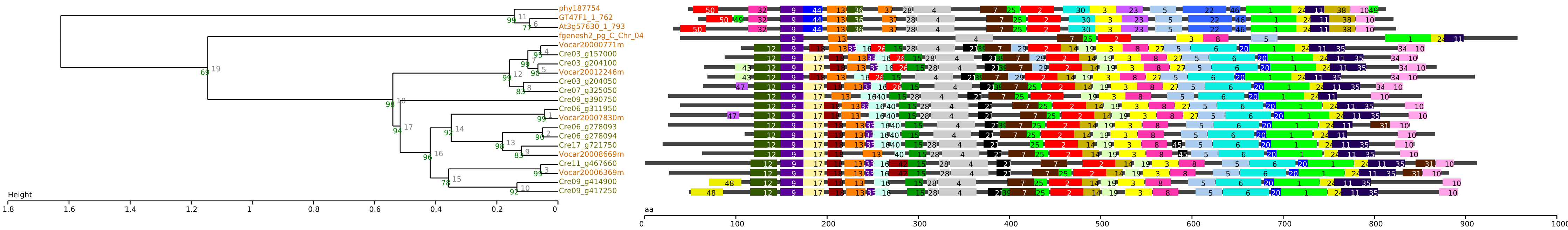

Supplementary Fig S8. SALAD analysis of chlorophyte and streptophyte full length EXAD and EXAD-like sequences. The EGF-like domains (Domain 4 and 9) are conserved as well as C-terminal domains putatively involved in the catalytic activity.

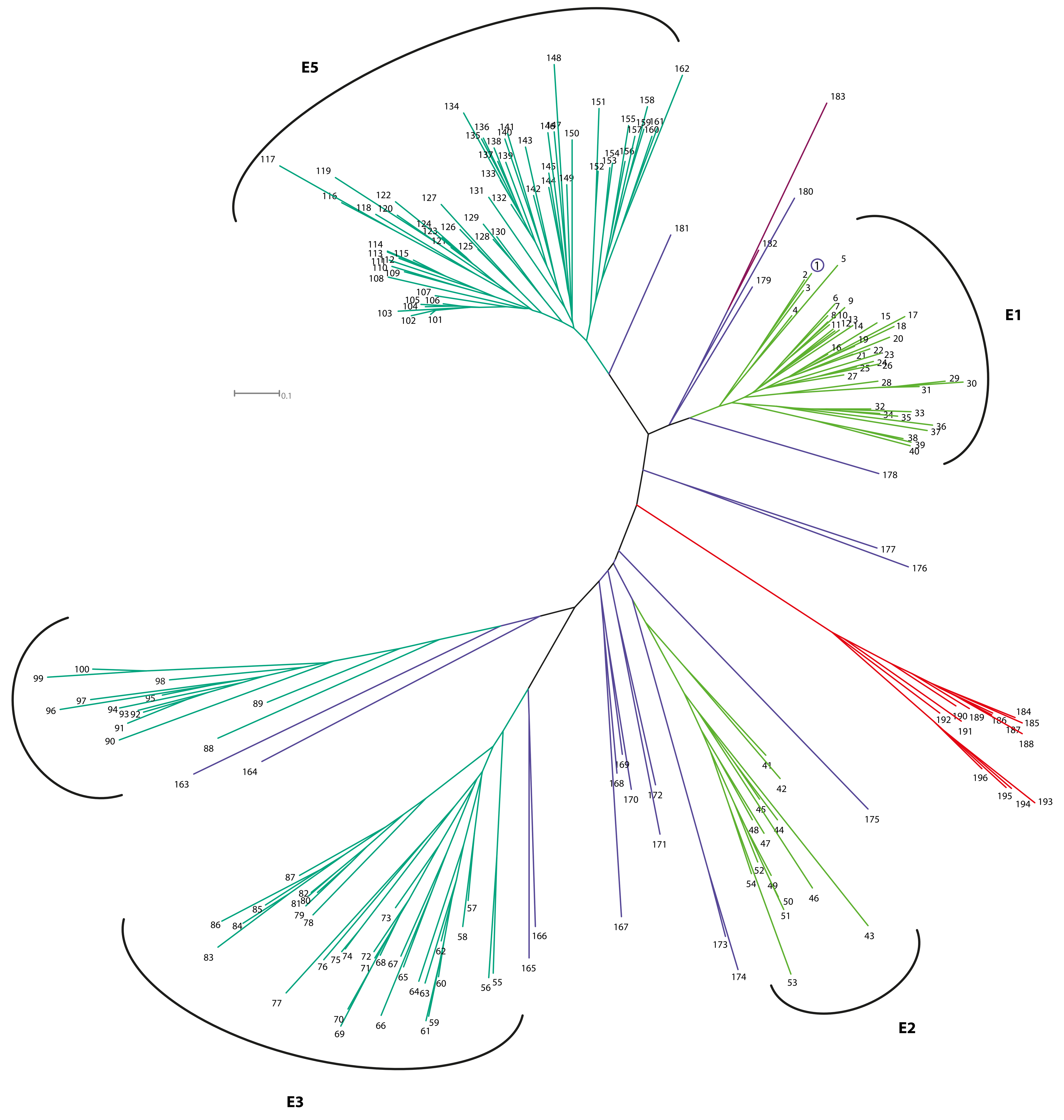

Supplemental figure S9. Key to the sequences used for building the phylogenetic tree in Fig. 7. Arabidopsis ExAD is number 1 (circled). The Newark tree file is provided as ExpandedCladeEnewarkTree.txt. The numbers refer to entries in the Excel sheet enclosed as ExpandedCladeEsequences.xlsx. The fasta headers in the spreadsheet refer to expandedEclade.fasta.

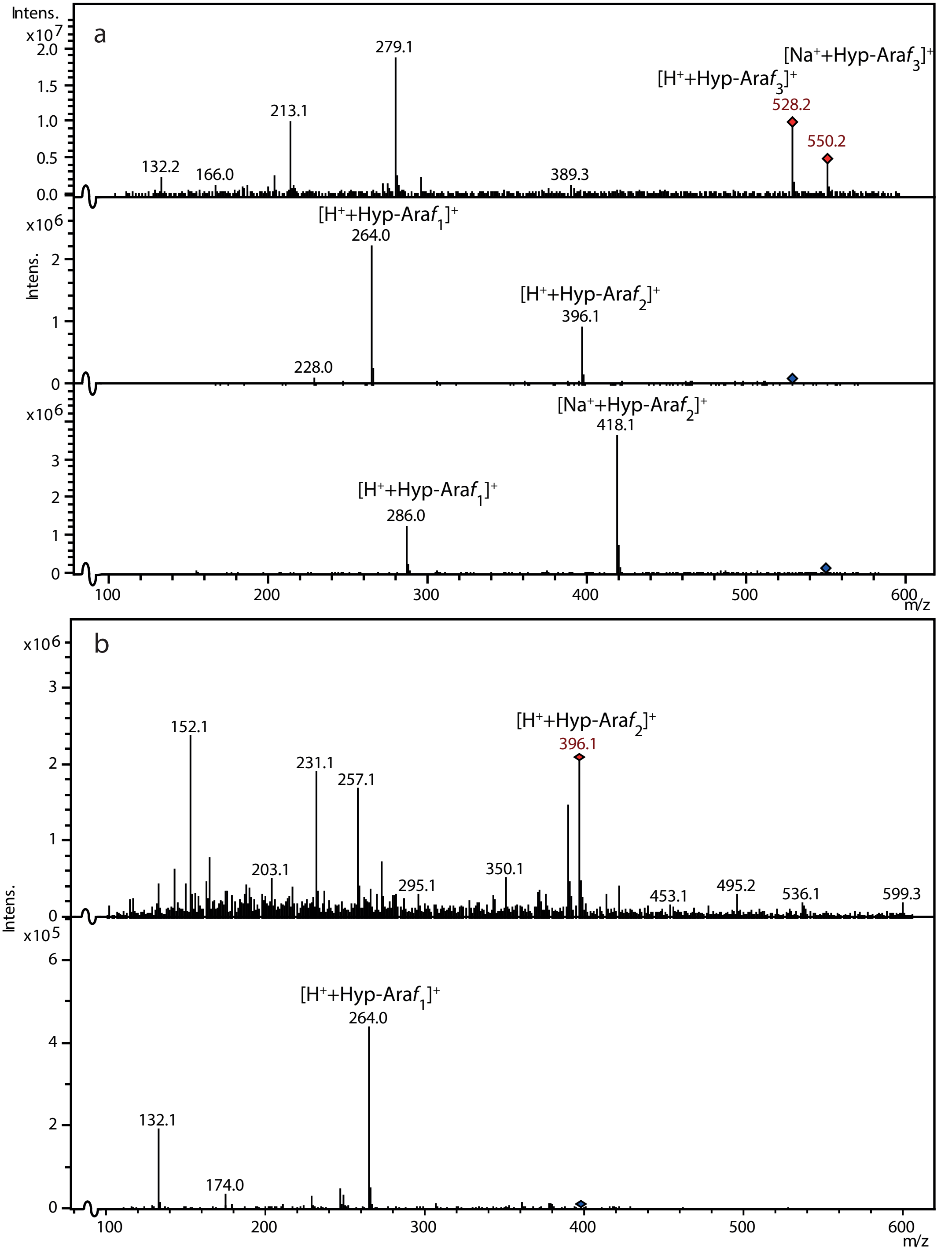

Supplementary figure S10. MS/MS spectrum of a barium hydroxide hydrolysate of *Klebsormidium accidum* AIR. MS/MS spectrum (direct inlet) of a barium hydroxide hydrolysate of *K. flaccidum* AIR demonstrationg the presence of Hyp-Pen$_3$/Hyp-Ara*f*$_3$ (*a*) and Hyp-Pen$_2$/Hyp-Ara*f*$_2$ (*b*).

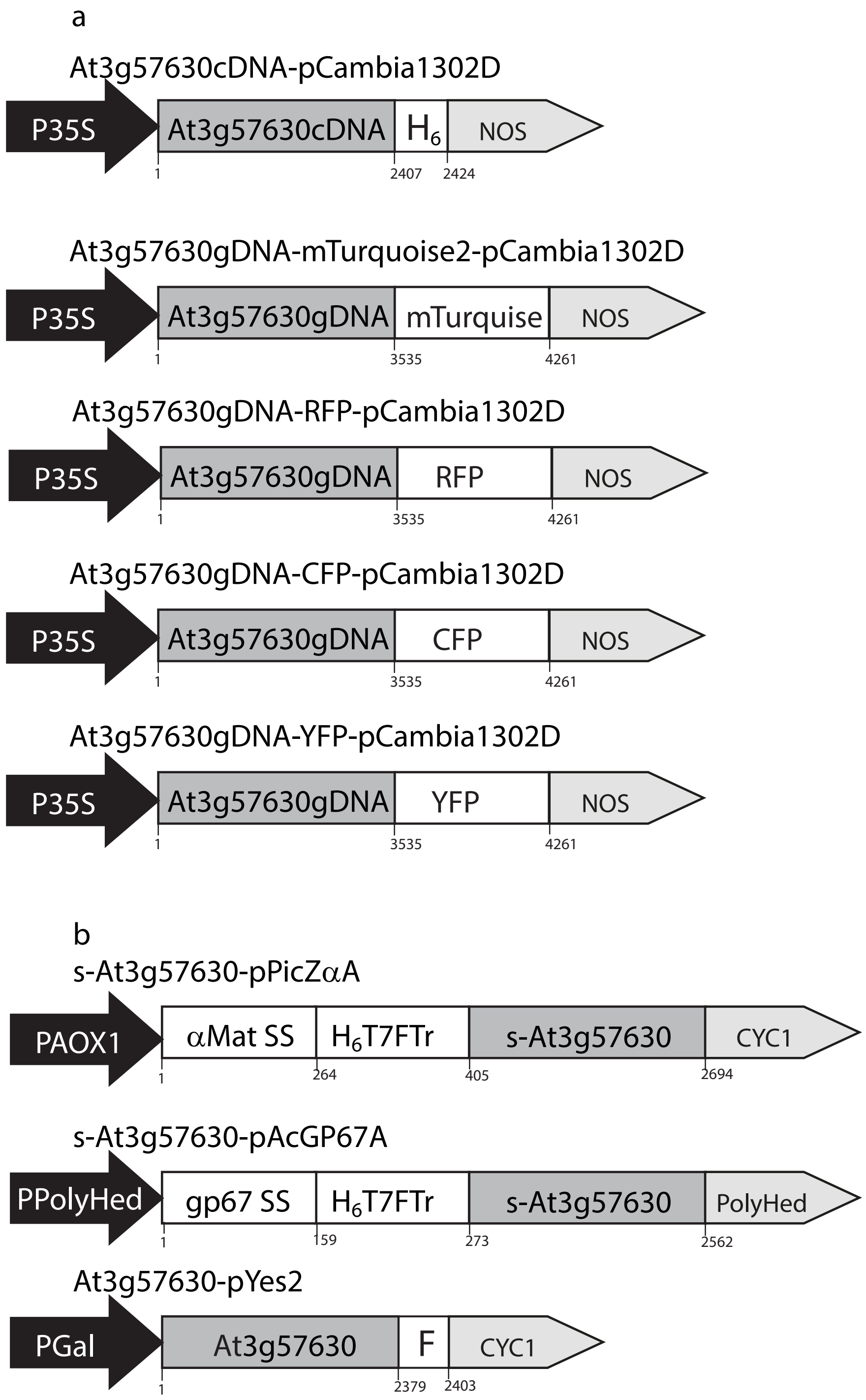

Supplementary Material and Methods figure S11. Construct designs. *a)* Constructs for in plant complementation, over expression and subcellular localization. *b)* Constructs for heterologous expression. s, F, H6-7, T7, c-Myc and Tr designate soluble, Flag tag (DYKDDDDK), poly Histidine$_{6\text{-}7}$ tag, T7-Tag (MASMTGGQQM), c-Myc tag (GS-EQKLISEEDL) and Trombin site (LVPRGS), respectively. Except for the intron containing gDNA construct, positions are given according to decduced amino acids. P35S, PAOX1, PPolyHed and PGal1 designate Cauliflower mosaic virus 35S (CaMV-35S) promotor, *Pichia pastoris* alcohol oxidase 1 (AOX1) promotor of pPicZαA (Invitrogen), baculovirus polyhedrin promoter of p*Ac*GP67A (Invitrogen) and *Saccharomyces cerevisiae* PGal1 promotor of pYES-2 (Invitrogen), respectively. NOS, CYC1 and PolyHed designate nopaline synthase, *Saccharomyces cerevisiae* cytochrome c-1 and baculovirus polyhedrin transcription terminators, respectively.

Supplementary Methods

Genotyping of *exad1-1* (SAIL_843_G12), *exad1-2* (SALK_206288C) and *exad1-3* (SALK_204414C)

Homozygous T2 lines were identified by PCR and RT-PCR analysis of the T2 generation of 10 dark grown etiolated seedlings. gDNA and total RNA were extracted from 10 dark grown etiolated seedlings, in 50 µl extraction buffer (20 mM Tris pH 8.0, 2.5 mM EDTA, 12.5 mM NaCl, 0.05 % SDS) (gDNA) and using an RNeasy Plant mini kit (Qiagen, Valencia, CA, USA) (total RNA) which were reverse-transcribed using the script cDNA Synthesis kit (Bio-Rad, Hercules, CA, USA) employing hexa nucleotide priming. PCR conditions (in total volumes of 50 µl) were as follows: Homozygous T2 lines were identified by PCR and RT-PCR analysis of the T2 generation of 10 dark grown etiolated seedlings. gDNA and total RNA were extracted from 10 dark grown etiolated seedlings, in 50 µl extraction buffer (20 mM Tris pH 8.0, 2.5 mM EDTA, 12.5 mM NaCl, 0.05 %SDS) (gDNA) and using an RNeasy Plant mini kit (Qiagen, Valencia, CA, USA) (total RNA) which were reverse-transcribed using the script cDNA Synthesis kit (Bio-Rad, Hercules, CA, USA) employing hexa nucleotide priming. PCR conditions were in a total volumes of 50 µl as follows: Gene (At3g57630) specific Left Primer (LP) & Right Primer (RP) and T-DNA specific Right Border Primer (RB) were 5'-ATTCAAATTTAAAATGCCGGC-3', 5'-TCGTTGCTTCCATGGATTTAC-3' and LB3 5'-TAGCATCTGAATTTCATAACCAATCTCGATACAC-3', for *exad1-1* (SAIL_843_G12); 5'-ATTCAAATTTAAAATGCCGGC-3', 5'-TCGTTGCTTCCATGGATTTAC-3'and LBb1.3 5'- ATTTTGCCGATTTCGGAAC-3' for *exad-2* (SALK_206288C) , 5'-AAATCCACATGACTCTGGCAC-3', 5'- TGTAAACCCATGACGTTTTCC-3' and 5'-ATTTTGCCGATTTCGGAAC-3' for *exad-3* (SALK_204414C) gDNA and 5'-TGAGGTGCATCATCAGCACG-3' and 5'- GCAATGTGGATCCAGAAGATGC-3' for *exad-1-3* cDNA. Primers for scoring ExAD insertion (*exad1-1-* ExAD cDNA complemented lines) were CLP 5'-CCAGACCCTTATAGCATGCG-3' and CRP 5'-GCCTTTTGTCTCTCGGCTTC-3'. Actin (At3g18780) primers were 5'-ACATTGTGCTCAGTGGTGGA-3' (Forward) and 5'-TCATACTCGGCCTTGGAGAT-3´ (Reverse) yielding bands of 280 and 208 bp on gDNA and cDNA, respectively. For LB3 and LBb1.3 primers (http://signal.salk.edu/tdnaprimers.2.html). PCR parameters for Actin: 98 °C for 10 min, followed by 40 cycles of 98 °C for 30 s, 60 °C for 30 s, and 72 °C for 30 s. 1 cycle of 72 °C for 10 min and hold at 4°C. ExAD cDNA: 95 °C for 4 min, followed by 30 cycles of 95 °C for 30 s, 65-55 °C gradient for 30 s, and 72 °C for 60 s. Hold at 4°C.

ExAD gDNA: 95 °C for 4 min, followed by 33 cycles of 95 °C for 30 s, 60 °C for 60 s, and 72 °C for 30 s. 1 cycle of 72 °C for 7 min and hold at 4°C.

Alpha-Arabinofuranosidase treatment

4 weeks old rosette leaves from *A. thaliana* were ground in in liquid N_2 (~500 mg ground powder) an processed (AIR, extensin rich fraction and $Ba(OH)_2$ treatment) as described above to be used for α-Arabinofuranosidase treatment assays: 250 μl of the hydrolysis product was used for α-Arabinofuranose assay in a 500 μl reaction with 1U/ml (2 μl) α-L-Arabinofuranosidase (novel specificity, *Bifidobacterium adolescentis,* Megazyme) and 50mM Ammonium formate, pH 6.0, incubated overnight at 37 °C.

The 500 μl assay reaction was purified with a Carbograph Extract-Clean 4 ml column (Alltech) as described[8]: the column was equilibrated by washing with 1.5 ml acetonitrile then 1.5 ml water, the assay mixture were applied allowing binding of the glycans, the followed by washing with 1.5 ml H_2O. The glycans were then eluted with 40% acetonitrile w/w H_2O in 500 μl fractions with elution in the first fraction, which was freeze dried and re dissolved in 50 μl H_2O, which was diluted 20 × in H_2O before analysis by ESI-MS.

Constructs for secreted expression of AT3G5760 in *Pichia pastoris* and baculo-virus High Five™ cells

Synthetic DNA sequence encoding AT3G57630, codon optimized for expression in *Pichia pastoris* and *High Five*™ cells, was obtained from Eurofins, Europe (http://www.eurofinsdna.com/ (sAT3G57630-pEX-A). The primer-set: Oli-1 (5' ATG<u>CACGTG</u>GGTCCAGTCGTTCCATCC-3) and Oli-10 (5' <u>GCGG*CCGC*</u>*GG*TTAGGAGGTCTT-3'), where underscoring designate *PmlI* and *NotI* restriction sites and italic designate *SacII* restriction sites, using AT3G57630-pEX-A, were used to PCR amplify the soluble part of AT3G5760 (sAT3G5760, aa 32-793). The PCR product was cloned into the pCR®2.1 vector according to the manufactors's protocol: TOPO TA Cloning Kit (Invitrogen No. K4500-1). The genes were excised from the pCR®2.1 vector using PmlI and SacII, and ligated into the pPicZαA vector, in frame with the α-mating factor[9] with a N-terminal His_6-, Thrombin (LVPRGS), Flag (DYKDDDDK) and T7 (MASMTGGQQM) (T7) tag yielding s-H_6T7FTr-sAt3g5760-pPicZαA.

H_6T7FTr-sAT3G5760 was excised from H_6-T7-F-Tr-sAt3g5760-pPicZαA using NotI (underlined) and cloned in frame with gp67 signal sequence in pAcGP67A Baculo virus Transfection vector, using *NotI*, thus bringing expression of H_6T7FTr-sAT3G5760 under control of the strong baculovirus polyhedrin promoter, yielding s-H_6T7FTr-sAt3g5760-pAcGP67A.

gDNA and total RNA extraction

gDNA and total RNA were extracted according to the manufacturers instruction using DNeasy kit (Qiagen) and RNeasy (Qiagen), respectively. 20 µl of Extracted RNA was treated with Ambion DNA-free™ DNA Removal Kit (ThermoFisher scientific cat. AM1906) according to the manufacturer instruction. 1 µg of RNA was reverse transcribed into cDNA using iScript™ cDNA Synthesis Kit according to the manufacturers instruction.

Constructs for expression of full length AT3G5760 in *Saccharomyces cerevisae*

ExAD was amplified from At3g57630-F-H_6-pCambia 1302D with the primers '5-*GAATTC*ACCATGGTTTCTCACCAGAAATG-3' and '5-*TCTAGA*TCA*CTTGTCATCATCGT*CCTTGTAGTCG-3' PCR was performed in 50 µl reaction volumes using the Expand High Fidelity system (Beohringer Ingelheim, Copenhagen, Denmark) with the cycle parameters: 3 min at 94 °C followed by 30 cycles of 30 sec at 95 ^{o}C, 30 sec at 58 ^{o}C and 3min at 72 ^{o}C followed by 5 min at 72 °C. PCR amplification was cloned into the pCR®2.1 vector using the TOPO-TA cloning kit (Invitrogen) giving PCR2.1-ExAD-F, with. ExAD-F was excised from PCR2.1 using EcoRI and XbaI (underlined), and cloned into pYES-2 using EcoRI and XbaI, thus bringing expression of EXAD-F under control of the Galactose inducible promoter, PGal1 in pYES-2 (Invitrogen).

Expression of T7-F-H-Tr-sAT3G57630 in *Pichia pastoris*

Transformation, identification of *T_7-F-H-Tr-sAT3G57630* expressing clones, and expression were essentially done as described[10], with expression (MeOH induction) volumes of 50 mL in 250 mL Elenheimer flasks and host strain KM71H (Invitrogen). Media fractions were dialyzed against 1 L 25 mM ammonium formate, pH 7.5, using MEdicell MWCO 12-14000 Da dialysis bags (KEBO Lab) and concentrated using a Amicon Ultra-4 Centrifugation filter (Milipore no. UFC803024). Pellet/precipitate formed in the bottom of the spin-filter was re-dissolved in 200 µl 25 mM ammonium formate, pH 7.5.

Per 100 µl cell pellet volume, 100 µl 50 mM NaH_2PO_4, pH 8, 300 mM NaCl, 10 mM imidazole and 200 µl glass beads (0.5 mm) were added, vortexed rigoursly, 10 min, 4 ^{o}C, spun 12000 × g 5 min, and the supernatant were subjected to arabinosyltransferase assays and western blot analysis.

Expression of T7-F-H-Tr-sAT3G57630 in insect High Five™ cells and NTA purification from spelt media

Transfection of *T7-F-H-Tr-sAT3G57630*-pAcGP67A and expression of *T7-F-H-Tr-sAT3G57630* in *High Five*™ cells were done as previously described[11,12]. 400 ml media fraction was dialysed at 4 °C in 5 L 25 mM Tris, pH 8, 300 mM NaCl (6 × normal NaCl concentration to prevent precipitation of *T7-F-H-Tr-sAT3G57630*) using MEdicell MWCO 12-14000 Da dialysis bag (KEBO Lab). A column of 2.5 ml of Ni-NTA beads (Qiagen no. 30210) was prepared and washed with water and equilibration buffer (35 mM Tris-HCl, pH 8, 300 mM NaCl, 10 mM imidazole). The ca. 500 ml dialysed media fraction was applied to the column, which was washed with wash buffer (25 mM Tris-HCl, pH 8, 300 mM NaCl, 20 mM imidazole) and protein was eluted in 13 × 1 ml fractions by elution buffer (25 mM Tris, pH 8, 300 mM NaCl, 300 mM imidazole).

Expression of AT3G57630-F in *Saccharomyces cerevisiae* and microsome isolation

Saccharomyces cerevisiae strain INVSc1 (MATa, his3D1, leu2, trp1-289, ura3-52, MAThis3D1, leu2, trp1-289, ura3-52) was transformed with pYES2-ExAD-F as described[13]. Yeast was grown in 10 ml culture of YPAD Glc (28 ºC, 150 rpm), spun down at 1700 × g and resuspended in 50 ml YPAD-gal (induction media) to a final OD_{600} of 0.4 and grown overnight at 28 °C, 150 rpm. Cells were pelleted at 1700 × g and resuspended in 1½ volume of homogenization buffer (29 % Glycerol, 0.1 M Tris-HCL (pH 7.5), 0.01 M EDTA, 0.05 M KCl, 1 mM DTT, 0.2 mM PMSF, 2 µg/ml pepstatin A. To this was added an equivalent volume of 0.5 mm glass beads. Cells were disrupted by vortexing at full speed five times 1 min followed by 1 min on ice. Homogenate was spun down at 3900 × g and supernatant was transferred to an ultracentrifugation tube. Microsomes were collected by spinning at 200000 × g for 1 hour and resuspended in 500 µl GTED (20 % glycerol, 0.1 M Tris-HCl (pH7.5), 1 mM EDTA, 0.05 M KCl, 1 mM DTT, 0.2 mM PMSF, 2 µg/µl pepstatin A).

SDS-PAGE Western Blotting

Samples were dissolved in SDS-loading sample buffer (1:5), boiled for 5–10 min, spun down and separated on SDS containing 4–12 % polyacrylamide Tricine gels (Invitrogen) using the MOPS-SDS system (Invitrogen) as running buffer. Proteins were transferred to a nitrocellulose membrane, 0.2 µm pore size (BioRad no. 162-0097) using wet electro blotting (settings: 200 V, 150 mA, 200 W, 50 min). Following transfer, the nitrocellulose membranes were washed 2 × Phosphate Buffered Saline (PBS), 2–3 min, blocked in 1 × PBS 5 % skimmed milk powder (SKP), 30 min, washed in 1 × PBS, pH 8.0, probed with primary antibodies obtained from (Sigma, Catalog no. F3165-1MG) diluted 1:1000 in PBS 5 % SKP overnight at 4 °C under mild shaking, washed in 1 × PBS, probed with secondary antibody obtained from (Dakolyt no. P0161) diluted 1:1000 in PBS, 5 % SKP and washed 3 × 3 min in

1 × PBS and finally placed in 1 × PBS. Chemi-luminiscence was monitored using the Super Signal West Dura Extended Duration Substrate (Pierce no. 34076), which was visualized on a BioSpectrum (UVP BioImaging Systems, Upland, California, USA)

Synthesis of uridine di-phosphate-β-L-Arabinofuranose (UDP-β-L-Araf)

UDP-β-L-Ara*f* was chemically synthesised using the schemes as outlined[14] starting from anomerically pure β-L-arabinofuranosyl-1-phosphate, which itself was prepared by a selective acid degradation of (2,3,5-tri-*O*-acetyl-α,β-L-arabinofuranosyl)-1-phosphate.

Enzyme assays

Standard assay conditions were 0.25 μl 1 M ammonium formate, pH 7.5 (10 mM final), 1 μl 4.6 mM UDP-beta-L-Ara*f* (ca. 0.23 mM final), 3 μl of enzyme, 0,25 μl 1 M $MnCl_2$ (10 mM final) and 15 μl acceptor $Ba(OH)_2$ mediated peptide backbone hydrolysis of mutant extensin) in a final volume of 20 μl, which was incubated for 2 H, 25 °C, where after 2 μl fresh enzyme and 1 μl 4.6 mM UDP-L-Ara*f* was added and incubated overnight at 25 °C. Assays were then cleared by centrifugation at 12000 × g, 2 min, and the supernatant subjected to ESI-MS analysis. Concentration of the pre and post assay acceptor (Hyp-Ara$f_{1\text{-}3}$) was unknown, but it was clearly identifiable by ESI-MS.

Assay setup for Matrix Assisted Laser Desorption Ionization Time of Flight (MALDI-TOF) mass spectroscopy was 0.25 μl 1 M ammonium formate, pH 7.5 (10 mM final), 40 μl 4.6 mM UDP-L-Ara*f* (ca. 23 mM final), 0.25 μl 1 M $MnCl_2$ (10 mM final), and 15 μl of Gf(Muc1-2.5TR-(Araf_3)p, and 3 μl enzyme a final volume of 41 μl. During the 4 hours incubation an additional 5 μl 4.6 mM UDP-beta-L-Ara*f* was added each hour and every two hours 2 μl enzyme fraction. The assay products were analyzed MALDI-TOF and Electrospray-Ionisation Mass Spectrometry (ESI-MS). Concentration of acceptor epitope (Hyp-Araf_3) on Gf(Muc1-2.5TR-(Araf_3)p was estimated to ca. 4 μM.

Purification of Gf(Muc1-2.5TR-(Araf$_n$)p for MALDI-TOF analysis

Post assay Gf(Muc1-2.5TR-(Araf_n)p subjected to endoproteinase Asp-N digestion, followed by Zip-tip purification, and analysed by MALDI-TOF and by ESI mass spectroscopy of $Ba(OH)_2$ mediated peptide backbone hydrolysis. Asp-N digestion: 2 μg of endoprotinase Asp-N (Roche no. 11054589001) dissolved in 100 μl 0.1 M Tris-HCl, pH 8, to a final concentration of ca. 0.02 μg/μl. To each 20 μl of assay, 5 μl of Asp-N solution and 75 μl of H_2O were added. Asp-N digests were incubated overnight, 37 °C. Zip-Tip purification: A Zip-Tip (C-18, Milipore no. ZTC18S096) was prepared by rinsing with 0.1 % trifluoacetic acid (TFA) in 100 % acetonitrile followed by 10 μl 0.1 % TFA in ddH_2O. The 100 μl asp-N

digest mix was applied to the zip-tip by pipetting the sample through the column three times, which was followed by washing with 0.5 % formic acid. The peptides were eluted from the Zip-tip with 7.5 μl 1:1 0.5 % formic acid (vol/vol) water : acetonitirile solution followed by 7.5 μl 100 % acetonitrile. The purified peptide, in 15 μl 2:1 acetonitrile and formic acid (vol/vol) H_2O, was used for MALDI-TOF analysis.

Matrix assisted laser desorption ionization – time of flight mass spectroscopy analysis

Samples were prepared for analysis by placing 0.5 μl of sample solution on a MALDI target, followed by 0.5 μl of matrix solution (2.5-dihydroxybenzoic acid (Sigma- Aldrich) dissolved (25 g/l) in a 1:1 mixture of ddH_2O and MeOH), which was then air-dried. Mass spectra were acquired in the linear mode on a Voyager-Elite MALDI-TOF mass spectrometer (Perseptive Biosystem Inc., Framingham, MA) equipped with delayed extraction; external calibration was used.

Supplementary References

1 Hruz, T. *et al.* Genevestigator v3: a reference expression database for the meta-analysis of transcriptomes. *Adv Bioinformatics* **2008**, 420747, doi:10.1155/2008/420747 (2008).

2 Aoki, Y., Okamura, Y., Tadaka, S., Kinoshita, K. & Obayashi, T. ATTED-II in 2016: A Plant Coexpression Database Towards Lineage-Specific Coexpression. *Plant Cell Physiol* **57**, e5, doi:10.1093/pcp/pcv165 (2016).

3 Esquerre-Tugaye, M. T., Lafitte, C., Mazau, D., Toppan, A. & Touze, A. Cell Surfaces in Plant-Microorganism Interactions: II. Evidence for the Accumulation of Hydroxyproline-rich Glycoproteins in the Cell Wall of Diseased Plants as a Defense Mechanism. *Plant Physiol* **64**, 320-326 (1979).

4 Shi, Y. *et al.* Influence of EARLI1-like genes on flowering time and lignin synthesis of Arabidopsis thaliana. *Plant Biology* **13**, 731-739, doi:10.1111/j.1438-8677.2010.00428.x (2011).

5 Gao, Y. *et al.* Auxin binding protein 1 (ABP1) is not required for either auxin signaling or Arabidopsis development. *Proceedings of the National Academy of Sciences* **112**, 2275-2280, doi:10.1073/pnas.1500365112 (2015).

6 Pitzschke, A., Xue, H., Persak, H., Datta, S. & Seifert, G. J. Post-Translational Modification and Secretion of Azelaic Acid Induced 1 (AZI1), a Hybrid Proline-Rich Protein from Arabidopsis. *Int J Mol Sci* **17**, doi:10.3390/ijms17010085 (2016).

7 Cecchini, N. M., Steffes, K., Schlappi, M. R., Gifford, A. N. & Greenberg, J. T. Arabidopsis AZI1 family proteins mediate signal mobilization for systemic defence priming. *Nat Commun* **6**, 7658, doi:10.1038/ncomms8658 (2015).

8 Packer, N. H., Lawson, M. A., Jardine, D. R. & Redmond, J. W. A general approach to desalting oligosaccharides released from glycoproteins. *Glycoconjugate journal* **15**, 737-747 (1998).

9 Brake, A. J. *et al.* Alpha-factor-directed synthesis and secretion of mature foreign proteins in Saccharomyces cerevisiae. *Proceedings of the National Academy of Sciences of the United States of America* **81**, 4642-4646 (1984).

10 Petersen, B. L. *et al.* Assay and heterologous expression in Pichia pastoris of plant cell wall type-II membrane anchored glycosyltransferases. *Glycoconjugate journal* **26**, 1235-1246, doi:10.1007/s10719-009-9242-0 (2009).

11 Egelund, J. *et al.* Arabidopsis thaliana RGXT1 and RGXT2 encode Golgi-localized (1,3)-alpha-D-xylosyltransferases involved in the synthesis of pectic rhamnogalacturonan-II. *Plant Cell* **18**, 2593-2607, doi:10.1105/tpc.105.036566 (2006).

12 Wandall, H. H. *et al.* The lectin domains of polypeptide GalNAc-transferases exhibit carbohydrate-binding specificity for GalNAc: lectin binding to GalNAc-glycopeptide substrates is required for high density GalNAc-O-glycosylation. *Glycobiology* **17**, 374-387, doi:10.1093/glycob/cwl082 (2007).

13 Gietz, R. D. & Schiestl, R. H. Quick and easy yeast transformation using the LiAc/SS carrier DNA/PEG method. *Nat Protoc* **2**, 35-37, doi:10.1038/nprot.2007.14 (2007).
14 Zhang, Q. & Liu, H.-w. Chemical synthesis of UDP-β-l-arabinofuranose and its turnover to UDP-β-l-arabinopyranose by UDP-galactopyranose mutase. *Bioorganic & Medicinal Chemistry Letters* **11**, 145-149, doi:http://dx.doi.org/10.1016/S0960-894X(00)00616-8 (2001).
